# Benzenesulphonamide inhibitors of the cytolytic protein perforin

**DOI:** 10.1016/j.bmcl.2016.12.057

**Published:** 2017-02-15

**Authors:** Julie A. Spicer, Christian K. Miller, Patrick D. O'Connor, Jiney Jose, Kristiina M. Huttunen, Jagdish K. Jaiswal, William A. Denny, Hedieh Akhlaghi, Kylie A. Browne, Joseph A. Trapani

**Affiliations:** a Auckland Cancer Society Research Centre, Faculty of Medical and Health Sciences, The University of Auckland, Private Bag 92019, Auckland 1142, New Zealand; b Maurice Wilkins Centre for Molecular Biodiscovery, A New Zealand Centre for Research Excellence, Auckland, New Zealand; c School of Pharmacy, Faculty of Health Sciences, University of Eastern Finland, P.O. Box 1627, FI-70211 Kuopio, Finland; d Cancer Immunology Program, Peter MacCallum Cancer Centre, 305 Grattan Street, Melbourne, Victoria 3000, Australia; e Sir Peter MacCallum Department of Oncology, The University of Melbourne, Parkville, Victoria 3052, Australia

**Keywords:** Perforin, Perforin inhibitor, Benzenesulphonamide, Bioisostere, Immunosuppressant

## Abstract

The pore-forming protein perforin is a key component of mammalian cell-mediated immunity and essential to the pathway that allows elimination of virus-infected and transformed cells. Perforin activity has also been implicated in certain auto-immune conditions and therapy-induced conditions such as allograft rejection and graft versus host disease. An inhibitor of perforin activity could be used as a highly specific immunosuppressive treatment for these conditions, with reduced side-effects compared to currently accepted therapies. Previously identified first-in-class inhibitors based on a 2-thioxoimidazolidin-4-one core show suboptimal physicochemical properties and toxicity toward the natural killer (NK) cells that secrete perforin *in vivo*. The current benzenesulphonamide-based series delivers a non-toxic bioisosteric replacement possessing improved solubility.

## Graphical abstract

**Figure:**
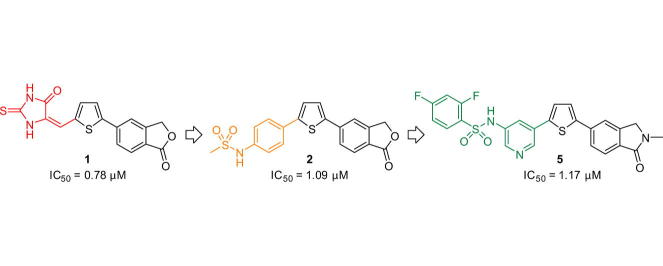

The primary role of cytotoxic T lymphocytes (CTL) and NK cells is to eliminate virally infected or oncogenic target cells.^1^ This process takes place *via* the granule exocytosis pathway^2^ where, upon stable conjugation with a target cell, the contents of cytotoxic granules contained in CTL or NK cells are secreted into the synaptic cleft formed between effector and target. This exposes the plasma membrane of the target cell to the combined action of perforin, a 60 kDa calcium-dependent member of the membrane-attack complex/perforin (MACPF) family of proteins, and a group of serine proteases known as granzymes. Perforin is a pore-forming protein and is essential for this process since it facilitates infiltration of the target cell by the granzymes, which in turn initiates apoptotic cell death. At high concentrations recombinant perforin is also cytolytic in its own right and the physiological means by which granzymes are delivered to the target cell cytosol has until recently been the subject of hot debate.^1^, ^2^, ^3^ But recent studies utilising time-lapse microscopy have shown that target cell death is invariably achieved through apoptosis (not lysis) and that perforin exerts its biological effects by causing transient osmotic disruption of the target cell plasma membrane, not endosomal vesicles. Accordingly, membrane perturbation by perforin pores is sufficient to permit direct diffusion of granzymes into the target cell.^4^ The process is remarkably rapid, with time-lapse microscopy revealing that perforin exocytosis and target cell permeabilisation takes place within 30 s, while pore repair is initiated and completed in another 80 s – sufficient time for the delivery of a lethal dose of granzymes.^4^ Perforin is composed of an N-terminal MACPF domain and an EGF-like central shelf, below which is located a membrane-interacting C2 domain.^5^ The protein binds efficiently to cell membranes in the absence of calcium but requires binding to become membranolytic.^6^, ^7^ Upon exposure to calcium, perforin undergoes a conformational change that allows it to assemble into highly ordered aggregates of 20–22 molecules where each monomer contributes two β-hairpins to a β-barrel which spans the plasma membrane.^5^, ^8^

Defective delivery and/or non-functional perforin within the granule exocytosis pathway is known to be associated with various human disorders including familial haemophagocytic lymphohistiocytosis (FHL), an inability to clear viral infections, and susceptibility to haematological malignancies.^3^ Inappropriate perforin activity has also been implicated in a variety of pathologies, including cerebral malaria, insulin-dependent diabetes, juvenile idiopathic arthritis and postviral myocarditis^9^, ^10^, ^11^ as well as therapy-induced conditions such as allograft rejection and graft versus host disease.^2^, ^12^, ^13^ Since perforin is expressed exclusively by CTL and NK cells it is possible that a selective inhibitor of this protein could be used to treat autoimmune diseases or therapy-induced conditions characterised by dysfunction of this pathway. Unlike current immunosuppression therapies which have a wide range of side-effects, an inhibitor that targets this mechanism could result in a potent immunosuppressive therapy with greatly reduced side-effects.

The original lead for this programme arose from a high-throughput screen of approximately 100,000 compounds,^14^ and following an extensive SAR study,^15^, ^16^ compound **1** (Fig. 1) was identified as one of the most potent inhibitors of recombinant perforin-induced lysis of labelled Jurkat T lymphoma cells.

This work showed that while a thiophene B-subunit resulted in a significant increase in activity, all variations explored as potential replacements for the 2-thioxoimidazolidin-4-one A-subunit were either less potent or extremely insoluble.^15^ Introduction of an isoindolinone C-subunit (in place of an isobenzofuranone) to give **1** gave greater potency (Jurkat IC_50_ = 0.51 μM) with improved solubility, however a major drawback for the entire series was variable levels of toxicity when whole NK cells were used to deliver a lytic dose of perforin.^16^ Although selected compounds were tested *in vivo* and found to be well-tolerated with appropriate pharmacokinetics for future efficacy experiments, it was eventually concluded that toxicity might still be observed in the immunocompromised mice required for an efficacy study. Replacement of the 2-thioxoimidazolidin-4-one also remained a priority as it contained a potentially reactive Michael acceptor and existed as an interconverting and inseparable mixture of *E*- and *Z*-isomers. These issues were in part overcome through the design and synthesis of a series of diarylthiophenes.^17^ Our rationale was that a substituted aryl A-subunit could replicate the pattern of hydrogen bond donors and acceptors presented by the 2-thioxoimidazolidin-4-one and presumably required for activity. We were successful in identifying sub-micromolar inhibitors of isolated perforin (e.g., **2**, **3**) of which several had activity against whole NK cells with minimal toxicity. One significant drawback of these highly aromatic structures was their insolubility, which ranged between 0.023 and 16 μg/mL making them unsuitable for further drug development. In order to improve potency and particularly solubility, we emulated a strategy employed by GSK workers,^18^ where a thiazolidinedione was replaced with a pyridyl-linked benzenesulphonamide (**4**) to give a potent, orally bioavailable inhibitor of phosphoinositide 3-kinase α (PI3Kα) and mammalian target of rapamycin (mTOR) with *in vivo* activity. Given that we had already successfully identified an aryl sulphonamide (**2**) as a replacement for the closely related thioxoimidazolidinone, this approach complemented our existing SAR and offered an opportunity to target more potent, soluble perforin inhibitors.

*Synthesis* – The 2-thioxoimidazolidin-4-one subunit (**A**) of **1** was replaced with a pyridine-3-yl-2,4-difluorobenzenesulphonamide which was linked through thiophene to a range of cyclic amides and indoles (**C**), giving compounds **5**–**18** (Table 1). To connect the C-subunits and thiophenes, Suzuki reactions were carried out for each halide and boronate pair to give target compounds **23**–**34** (Scheme 1).

Selective iodination of the thiophene 2-position was then carried out with N-iodosuccinimide for compounds **35**–**45** and **48**, while for the indole targets (**16**, **17**) the use of 2,5-dibromothiophene in a Suzuki reaction gave the required intermediates with the bromide already in place (**46**, **47**). A second Suzuki step allowed the introduction of either the free amino or BOC-protected pyridyl-3-amine subunit to compounds **49**–**62**. Where required (**55**–**62**) the carbamate was deprotected using a 1:1 mixture of trifluoroacetic acid in CH_2_Cl_2_ to give amines **63**–**70**. Finally, reaction of the amines with 2,4-difluorobenzenesulphonyl chloride at low temperature gave the target products **5**–**18**. In several cases the bis-benzenesulphonamide was also formed; these were hydrolysed back to the mono-sulphonamide by stirring with a 1:1 mixture of 1,4-dioxane and 2 M NaOH at room temperature.

A small set of analogues where the thiophene of compound **5** was replaced with other heterocycles was also prepared (compounds **19**–**22**, Table 2 and Scheme 2).

For targets **19** and **20**, the 2-furan- and 2-thiazoleboronic acids were coupled with 5-bromo-2-methylisoindolin-1-one under standard conditions to give intermediates **71** and **72**, which were iodinated, coupled with pyridyl-3-amine-5-boronate, and reacted with 2,4-difluorobenzenesulphonyl chloride as previously described (Scheme 2).

Unfortunately the isomeric thiazole **21** could not be prepared in this manner as the attempted coupling of the thiazole bromide **77** and the pyridyl-3-amine boronate failed. In order to circumvent this problem, the pyridine-sulphonamide **78** was introduced as a single subunit *via* a Suzuki reaction. Protection of the sulphonamide NH with an ethoxymethyl group was required for a successful coupling and this was removed under acidic conditions to furnish the desired thiazole **21**. Finally, the pyridyl analogue **22** was prepared through reaction of 5-(4,4,5,5-tetramethyl-1,3,2-dioxaborolan-2-yl)pyridin-2-amine and 5-bromo-2-methylisoindolin-1-one to give the aminopyridine intermediate **79** (Scheme 3).

The amine was diazotized with NaNO_2_ in HBr, treated with Br_2_ to convert to the bromide **80**, then subjected to the same steps as the other compounds of Scheme 2 to give the final compound **22**.

*Structure–activity relationships:* Our strategy involved linking a pyridine-3-yl-2,4-difluorobenzenesulphonamide subunit *via* a thiophene to a variety of bicyclic templates A-N (Table 1). These templates were selected as those most likely to display activity, having been previously optimised in our thioxoimidazolidinone lead series.^16^ In the present case, we elected to concentrate on isoindolinone-based analogues rather than the isobenzofuranones we had studied previously due to the tendency of the lactone to ring-open in the presence of human plasma.^17^ Thus the first compound to be targeted was the N-methylisoindolinone **5**, the benzenesulphonamide analogue of the most potent compound in the thioxoimidazolidinone series (**1**). Gratifyingly, the activity of this compound (IC_50_ = 1.17 μM) was only ∼2-fold less than **1** (0.51 μM), demonstrating that it is possible to replace the thioxoimidazolidinone with a benzenesulphonamide moiety and retain potency.

The *N*H-isoindolinones **6** and **7** also showed appreciable activity (IC_50_s = 6.87 and 4.15 μM), although the isomeric N-methylisoindolinone **8** was ∼10-fold poorer. In contrast to the thioxoimidazolidinone series, for the benzenesulphonamides the 6-membered cyclic amides **9**–**12** all showed poor activity, as did the methyl-substituted analogue **13**. Given the activity displayed by the 5-membered examples **5**–**7**, we also elected to explore the isomeric oxindoles **14** and **15**. These too demonstrated significant activity with the *N*H-oxindole **14** (IC_50_ = 5.18 μM) ∼2-fold superior to its N-methyl partner **15** (10.3 μM). Finally, indoles **16**–**18** were investigated. Targets **16** and **17** showed good activity with IC_50_s = 5.80 and 7.58 μM respectively, however acetyl-substitution at the 3-position of the indole (**18**) was detrimental to activity. Overall, the N-methylisoindolinone-containing **5** was clearly the most potent compound which is consistent with our findings in the thioxoimidazolidinone series.

On the assumption that the SAR of the B-subunit would also follow that of the thioxoimidazolidinones, we selected a small set of heterocyclic replacements for the thiophene subunit based on our previous work (Table 2).^15^

Our objectives here were to both improve the solubility of the target compounds as well as mitigate any potential metabolic risk presented by the thiophene.^19^ As anticipated, introduction of a furan resulted in a loss of activity from IC_50_ = 1.17 μM to 15.0 μM. For the remaining thiazole (**20**, **21**) and pyridyl (**22**) analogues the activity was unexpectedly disappointing (IC_50_s = 17.1, >20, 8.17 μM respectively). In our historic series these heterocycles possessed activity similar to the lead thiophene, however, in the present case the predicted activity did not eventuate, suggesting that the SAR is tighter for this class of compounds.

*Biological activity, solubility & stability:* The most potent inhibitors of recombinant perforin-mediated lysis of labelled Jurkat cells were then tested for their ability to block whole NK cells from lysing ^51^Cr-labelled (K562 leukaemia) target cells (Table 3).

The use of NK cells to secrete perforin rather than employing isolated recombinant protein is a more stringent test of a compound’s ability to block lytic activity, requiring the inhibitor to access the synaptic cleft between effector (NK) and target cells where perforin is released. In this assay, inhibitor is co-incubated with KHYG-1 NK cells in medium for 30 min at room temperature, ^51^Cr-labelled target cells added and cell lysis evaluated after 4 h incubation at 37 °C by measuring ^51^Cr release. The viability of the NK cells in the presence of inhibitor was also assessed 24 h later to confirm that any inhibitory activity exhibited by the compounds was due to blocking the action of perforin and not non-specific killing of the effector cells.

Exemplars **5**, **6**, **7** and **14** from Table 1 were therefore selected as potent inhibitors of recombinant perforin. Indole **16** (IC_50_ = 5.80 μM) was excluded from this analysis due to its activity being adversely affected in the presence of serum. When **16** was tested at 20 μM concentration in a Jurkat assay containing 10% mouse serum, inhibitory activity was reduced from 88% (0.1% BSA alone) to 32%. This phenomenon has been observed in previous series we have studied,^15^, ^16^ however in the cases of **5**, **6**, **7** and **14** the activity in the presence of 10% mouse serum was similar to that obtained in its absence.

When tested for their ability to block NK cell-induced lysis, the four preferred compounds showed excellent levels of inhibition (80–91%) at a concentration of 20 μM and significantly, there was virtually no effect on NK cell viability (93–96% survival at 20 μM). This is an important result in the context of this work because we have observed varying degrees of toxicity toward the KHYG-1 cells in all the other series we have studied to date.^15^, ^16^, ^17^

Preliminary physicochemical data was also collected on **5**, **6**, **7** and **14** (Table 4) in order to assess their potential for progression to *in vivo* pharmacokinetic studies.

The N-methylisoindolinone **5** showed moderate solubility (1.1 mg/mL) and stability in solution (82% over 24 h) and was stable in the presence of mouse (92%) and rat (99%) microsomes, but some instability was observed for human microsomes (58%). The two *N*H-isoindolinones **6** and **7** showed excellent solubility, at 10.6 and 12.9 mg/mL respectively. Compound **6** also showed moderate stability in aqueous solution and good stability over all three species of microsomes (92, 88, 85%). In contrast, the isomeric isoindolinone **7** showed excellent stability in water (97%), rat and human microsomes but was metabolised extensively in the presence of mouse microsomes (42% remaining after 0.5 h). The oxindole **14** was stable in solution and in the presence of mouse, rat and human microsomes but possessed disappointing solubility at 0.39 mg/mL. Finally, all four compounds showed very high levels of plasma protein binding.

Given that the pyridine-3-yl-2,4-difluorobenzenesulphonamide subunit is derived from a structure that was optimised to target PI3Kα, it was also considered prudent to counter-screen for off-target effects related to this pathway. To that end, compound **5** was included in a high-throughput screen to test for PI3Kα activity (for details see Supplementary Data) where it was found that the IC_50_ was >6.25 μM. Thus any activity that lead compound **5** possesses toward PI3Kα is a minimum of 6-fold less than that observed against perforin. While this result still requires more detailed investigation, it at least provides some reassurance that these compounds are optimised as potent inhibitors of the desired target perforin rather than PI3Kα.

In previous studies we sought to design and optimise potent and “drug-like” inhibitors of perforin based on an initial hit from a mass screen. The most difficult obstacle to overcome proved to be replacement of a problematic 2-thioxoimidazolidinone moiety containing a potential Michael acceptor and showing variable toxicity toward the perforin-producing NK cells. Our overall approach is based on selectively inhibiting perforin to facilitate allograft transplantation, while at the same time preserving the NK cells so that withdrawal of the drug would be expected to restore NK cell killing within hours. Toxicity exhibited toward the NK cells is therefore an issue for both *in vivo* efficacy investigations and any potential clinical studies. In the current series the 2,4-difluorobenzenesulphonamide has been successfully employed as a bioisosteric replacement for the 2-thioxoimidazolidinone to give compounds with activity comparable to the best of our previous series. In addition, all four of the most potent inhibitors (**5**, **6**, **7**, **14**) showed >90% viability of the NK cells, a greater rate of survival than all preceding series and essentially non-toxic.

Compound **1** was the most potent inhibitor of perforin, while **6** had the best overall balance of solubility and stability. Although we had previously identified a set of diarylthiophene alternatives to the 2-thioxoimidazolidinones, these were extremely insoluble compared to the current series which shows far superior solubility. Although no single compound possessed an ideal profile, the overall combination of potency, solubility, non-toxicity and selectivity obtained in this study gives us confidence that further refinement will furnish an appropriate candidate for *in vivo* studies. For example, one area for further exploration will be whether it is possible to further modulate activity and physicochemical properties through substitution on the benzene ring of the sulphonamide. Our future objectives will therefore be to retain the benefits of the 2,4-difluorobenzenesulphonamide at the same time as further optimising this highly promising series of perforin inhibitors toward a potential clinical candidate.

## Figures and Tables

**Fig. 1:**
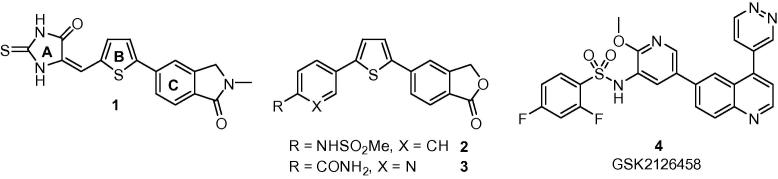
Historical inhibitors of perforin and PI3Kα clinical candidate GSK2126458

**Scheme 1:**
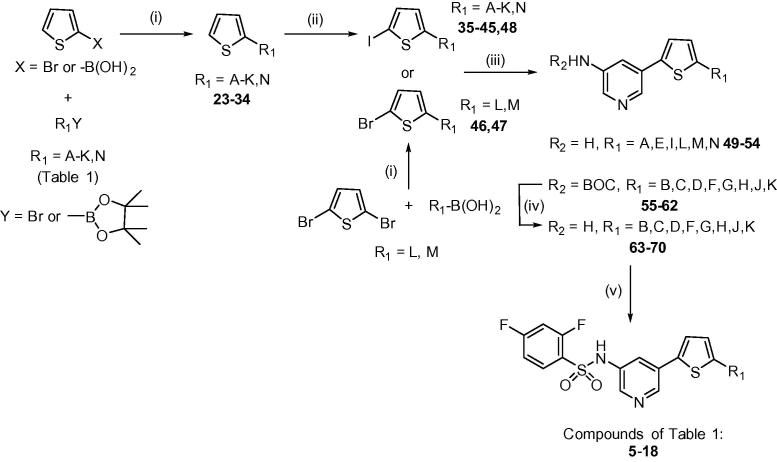
Reagents and conditions: (i) Pd(dppf)Cl_2_, EtOH/toluene, 2 M Na_2_CO_3_, reflux; (ii) NIS, AcOH, CHCl_3_, RT; (iii) 5-(4,4,5,5-tetramethyl-1,3,2-dioxaborolan-2-yl)pyridin-3-amine, *tert-*butyl (5-(4,4,5,5-tetramethyl-1,3,2-dioxaborolan-2-yl)pyridin-3-yl)carbamate or 3-aminopyridine-5-boronic acid, Pd(dppf)Cl_2_, EtOH/toluene, 2 M Na_2_CO_3_, reflux; (iv) TFA, CH_2_Cl_2_ (1:1), RT; (v) 2,4-difluorobenzenesulfonyl chloride, pyridine, 0–45 °C.

**Scheme 2:**
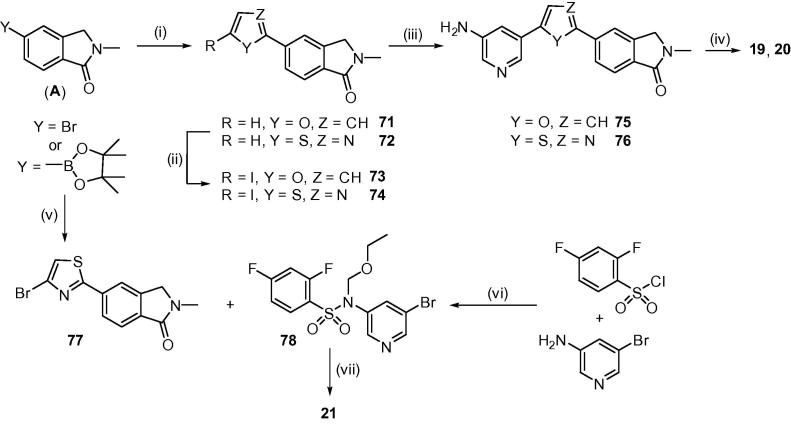
Reagents and conditions: (i) 2-furanboronic acid or 2-bromothiazole, Pd(dppf)Cl_2_, EtOH/toluene, 2 M Na_2_CO_3_, reflux; (ii) NIS, AcOH, CHCl_3_, RT; (iii) 5-(4,4,5,5-tetramethyl-1,3,2-dioxaborolan-2-yl)pyridin-3-amine, Pd(dppf)Cl_2_, EtOH/toluene, 2 M Na_2_CO_3_, reflux; (iv) 2,4-difluorobenzenesulfonyl chloride, pyridine, 0–45 °C; (v) 2,4-dibromothiazole, Pd(dppf)Cl_2_, EtOH/toluene, 2 M Na_2_CO_3_, reflux; (vi) a. Pyridine, 0–45 °C, b. (chloromethoxy)ethane, NaH, DMF, RT; (vii) a. **78**, bis(pinacolato)diboron, KOAc, Pd(dppf)Cl_2_, DMSO, 90 °C, 3 h; b. **77**, Pd(dppf)Cl_2_, EtOH/toluene, 2 M Na_2_CO_3_, reflux; c. 1:1 3 M HCl and 1,4-dioxane, reflux, 1 h, followed by 2 M NaOH to precipitate the sodium salt.

**Scheme 3:**
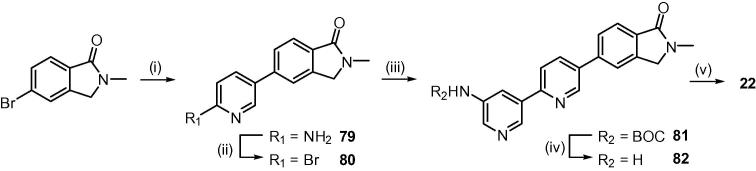
Reagents and conditions: (i) 5-(4,4,5,5-tetramethyl-1,3,2-dioxaborolan-2-yl)pyridin-2-amine, Pd(dppf)Cl_2_, EtOH/toluene, 2 M Na_2_CO_3_, reflux; (ii) a. 47% HBr, NaNO_2_, −10 °C; b. Br_2_, −10 °C-RT; (iii) *tert-*butyl (5-(4,4,5,5-tetramethyl-1,3,2-dioxaborolan-2-yl)pyridin-3-yl)carbamate, Pd(dppf)Cl_2_, EtOH/toluene, 2 M Na_2_CO_3_, reflux; (iv) TFA, CH_2_Cl_2_ (1:1), RT; (v) 2,4-difluorobenzenesulfonyl chloride, pyridine, 0–45 °C.

**Table 1:** Variation of the C-subunit.

**Figure:**
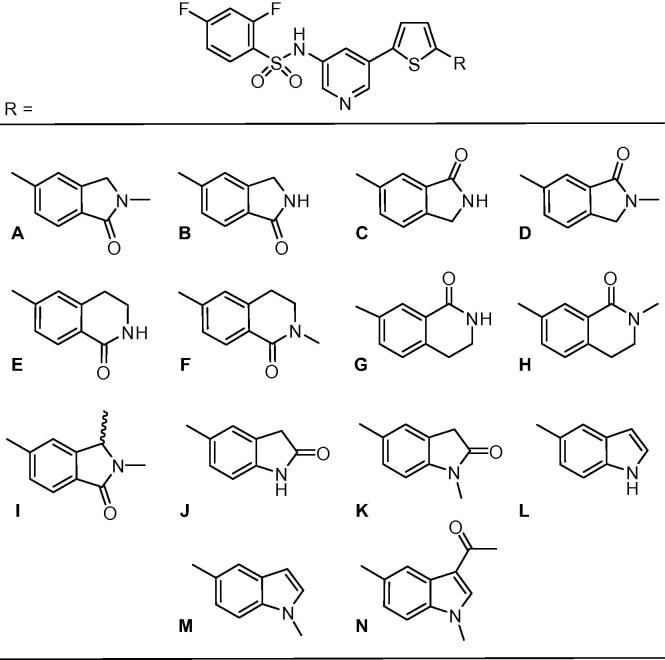

Number	R	Inhibition of Jurkat cell lysis IC_50_^a^ (μM)
**5**	A	1.17
**6**	B	6.87
**7**	C	4.15
**8**	D	10.4
**9**	E	>20
**10**	F	>20
**11**	G	>20
**12**	H	13.6
**13**	I	>20
**14**	J	5.18
**15**	K	10.3
**16**	L	5.80
**17**	M	7.58
**18**	N	11.2

a Testing was carried out over a range of doses, with the IC_50_ being equal to the concentration at which 50% inhibition of the lysis of Jurkat cells by perforin was observed, as measured by ^51^Cr release. Values are the average of at least two independent IC_50_ determinations.

**Table 2:** Thiophene replaced with other heterocycles.

**Figure:**
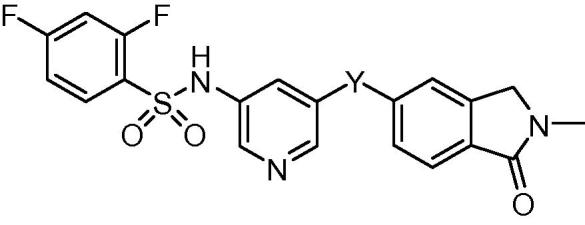

Compound	Y	Inhibition of Jurkat cell lysis IC_50_^a^ (μM)
**5**		1.17
**19**		15.0
**20**		17.1
**21**		>20
**22**		8.17

a Testing was carried out over a range of doses, with the IC_50_ being equal to the concentration at which 50% inhibition of the lysis of Jurkat cells by perforin was observed, as measured by ^51^Cr release. Values are the average of at least two independent IC_50_ determinations.

**Table 3:** Capacity of selected compounds to inhibit perforin delivered by KHYG-1 NK cells.

Number	Jurkat IC_50_ (μM)^a^	KHYG-1 inhibition (% at 20 μM)^b^	KHYG-1 viability (% at 20 μM)^c^
**5**	1.17	88 ± 3.8	93 ± 3.3
**6**	6.87	86 ± 1.3	94 ± 4.0
**7**	4.15	91 ± 1.3	96 ± 6.0
**14**	5.18	80 ± 13.1	96 ± 1.0

a Data given for the most potent compounds as determined by the Jurkat assay.

b Inhibition by compound (20 μM) of the perforin-induced lysis of ^51^Cr-labelled K562 leukemia target cells when co-incubated with KHYG-1 human NK cells. Percent inhibition calculated compared to untreated control (n = 4).

c Viability of KHYG-1 NK cells after 24 h by Trypan blue exclusion assay (n = 3). All results are the average of at least three separate determinations ± SEM (see Supplementary Data for details).

**Table 4:** Physicochemical properties of selected compounds.

Number	Solubility^a^ (μg/mL)	Stability in solution^b^ (%)	Microsome stability (%)^c^			Plasma protein binding^d^ (%)
			Mouse	Rat	Human	
**5**	1080	81.5	92	99	58	99.89
**6**	10,565	82.0	92	88	85	99.70
**7**	12,940	97.3	42	82	83	99.97
**14**	392	88.7	84	79	71	99.91

a Solubility of the sodium salt in water at room temperature; conversion to salt as described in General Procedure D (Supplementary Data).

b Percentage of parent compound (sodium salt) remaining after 24 h at 20 °C in water.

c Percentage of parent compound (sodium salt) remaining after exposure to mouse, rat or human microsomes for 30 min.

d Percentage of parent compound bound to mouse plasma after 6 h incubation at 37 °C as determined by equilibrium dialysis.
