# Supplementary material for: Benzenesulphonamide inhibitors of the cytolytic protein perforin
Source: Bioorg Med Chem Lett. 2017 Feb 15;27(4):1050–4. doi: 10.1016/j.bmcl.2016.12.057 (PMC5303009; doi:10.1016/j.bmcl.2016.12.057)
Supplement: Supplementary data 1 [file mmc1.docx]

**SUPPLEMENTARY DATA**

**Benzenesulphonamide Inhibitors of the Cytolytic Protein Perforin**

Julie A. Spicer^*^, Christian K. Miller, Patrick D. O’Connor, Jiney Jose, Kristiina M. Huttunen, Jagdish K. Jaiswal, William A. Denny, Hedieh Akhlaghi, Kylie A. Browne, Joseph A. Trapani

*^*^Corresponding author:* Tel.: +64 9 3737599. E-mail: j.spicer@auckland.ac.nz

**CONTENTS**:

1. Experimental Page 2

1.1. Chemistry Page 2

1.1.1. Table 1: Elemental analysis results for target compounds Page 29

1.1.2. Table 2: HRMS and HPLC results for target compounds Page 29

1.2. Biology Page 30

1.2.1 Inhibition of perforin-mediated lysis of Jurkat cells Page 30

1.2.2 KHYG-1 Cytotoxicity Assay Page 30

1.2.3 Toxicity to KHYG-1 NK Cells Page 31

1.2.4. PI3K-α Counterscreening Page 31

2. References Page 31

**1. Experimental**

**1.1. Chemistry**

Analyses were performed by the Microchemical Laboratory, University of Otago, Dunedin, NZ. Melting points were determined using an Electrothermal Model 9200 and are as read. NMR spectra were measured on a Bruker Advance 400 MHz spectrometer and referenced to Me_4_Si. Mass spectra were recorded either on a Varian VG 7070 spectrometer at nominal 5000 resolution or a Finnigan MAT 900Q spectrometer. All final compound purities were determined to be >95% by HPLC on an Alltech Alltima C18 column (3.2 x 150 mm, 5 μm) eluting with 5–80% MeCN/45 mM NH_4_HCO_3_.

**General procedure A: 2-Methyl-5-(thiophen-2-yl)isoindolin-1-one (23) (Scheme 1)**. 5-Bromo-2-methylisoindolin-1-one (520 mg, 2.30 mmol) and thiophene-2-boronic acid (442 mg, 3.45 mmol) were dissolved in a mixure of toluene (12 mL) and EtOH (6 mL). A solution of 2 M Na_2_CO_3_ (3 mL) and Pd(dppf)Cl_2_ (94 mg, 0.12 mmol) were added and the entire mixture heated at reflux under N_2_ for 2 h. Additional thiophene-2-boronic acid (294 mg, 2.30 mmol) was added and reflux continued under N_2_ overnight. Upon cooling, the mixture was diluted with water (100 mL) and extracted with CH_2_Cl_2_ (6x50 mL). The combined organic fractions were dried (Na_2_SO_4_), filtered, and the solvent removed under reduced pressure to give a crude solid which was purified by flash column chromatography on silica gel (EtOAc as eluant). The title compound was isolated as a light-brown solid (510 mg, 97%). ^1^H NMR [400 MHz, (CD_3_)_2_SO] δ 7.87 (s, 1 H), 7.77 (dd, *J* = 7.9, 1.6 Hz, 1 H), 7.67 (dd, *J* = 7.9, 0.3 Hz, 1 H), 7.61-7.66 (m, 2 H), 7.18 (dd, *J* = 5.0, 1.4 Hz, 1 H), 4.49 (s, 2 H), 3.08 (s, 3 H). LRMS (APCI^+^) calcd for C_13_H_12_NOS 230 (MH^+^), found 230.

**General procedure B: 5-(5-Iodothiophen-2-yl)-2-methylisoindolin-1-one (35)**. 2-Methyl-5-(thiophen-2-yl)isoindolin-1-one (**23**) (510 mg, 2.22 mmol) was dissolved in a mixture of CHCl_3_/AcOH (3:1, 16 mL) and *N*-iodosuccinimide (500 mg, 2.22 mmol) added. This mixture was stirred at room temperature for 2 h., then additional *N*-iodosuccinimide (500 mg, 2.22 mmol) added and stirring continued overnight. The reaction mixture was diluted with water (100 mL) and extracted with CH_2_Cl_2_ (3x50 mL). The combined CH_2_Cl_2_ fractions were washed with sat. NaHCO_3_ (2x50 mL), 1 M Na_2_S_2_O_5_ solution (50 mL), dried (Na_2_SO_4_) and filtered. The solvent was removed under reduced pressure to afford a solid which was triturated with Et_2_O and collected by filtration to give the title compound as a pale brown crystalline solid (693 mg, 88%). ^1^H NMR [400 MHz, (CD_3_)_2_SO] δ 7.82 (br s, 1 H), 7.71 (br d, *J* = 8.0 Hz, 1 H), 7.67 (d, *J* = 8.0 Hz, 1 H), 7.41 (d, *J* = 3.8 Hz, 1 H), 7.37 (d, *J* = 3.8 Hz, 1 H), 4.49 (s, 2 H), 3.07 (s, 3 H). LRMS (APCI^+^) calcd for C_13_H_11_INOS 356 (MH^+^), found 356.

**5-(5-(5-Aminopyridin-3-yl)thiophen-2-yl)-2-methylisoindolin-1-one (49).** 5-(5-Iodothiophen-2-yl)-2-methylisoindolin-1-one (**35**) was reacted with 5-(4,4,5,5-tetramethyl-1,3,2-dioxaborolan-2-yl)pyridin-3-amine (1.2 equivalents) according to general procedure A. The desired product was collected by filtration from the cooled reaction mixture and washed with H_2_O, MeOH and CH_2_Cl_2_. No further purification was required and the title compound was isolated as a green solid (84%), mp (MeOH/CH_2_Cl_2_) 244-248^o^C. ^1^H NMR [400 MHz, (CD_3_)_2_SO] δ 8.12 (d, *J* = 2.0 Hz, 1 H), 7.92 (s, 1 H), 7.90 (d, *J* = 2.5 Hz, 1 H), 7.80 (dd, *J* = 8.0, 1.3 Hz, 1 H), 7.70 (d, *J* = 4.3 Hz, 1 H), 7.68 (s, 1 H), 7.54 (d, *J* = 3.8 Hz, 1 H), 7.16 (t, *J* = 2.2 Hz, 1 H), 5.51 (br s, 2 H), 4.51 (s, 2 H), 3.09 (s, 3 H).

**General procedure C: 2,4-Difluoro-*N*-(5-(5-(2-methyl-1-oxoisoindolin-5-yl)thiophen-2-yl)pyridin-3-yl)benzenesulfonamide (5).** To 5-(5-(5-Aminopyridin-3-yl)thiophen-2-yl)-2-methyl- isoindolin-1-one (**49**) (225 mg, 0.79 mmol) in dry pyridine (23 mL) under N_2_ at RT, was added dropwise, 2,4-difluorobenzenesulphonyl chloride (336 mg, 1.58 mmol) in CH_2_Cl_2_ (3 mL) over 5 min. The suspension was heated to 45^o^C under N_2_ for 4 h., at which point another portion of 2,4-difluorobenzenesulphonyl chloride (169 mg, 0.79 mmol) in CH_2_Cl_2_ (2 mL) was added. The whole mixture was left to stir for at 45^o^C under N_2_ for 16 h., then the solvent removed under reduced pressure. The resulting residue was suspended in acetone (10 mL), 1 M HCl (20 mL) added, and the entire mixture stirred for 10 minutes. The solid was then collected by filtration, washed well with 1 M HCl and water, dried, and purified by chromatography as described below.

In cases where the bis-sulphonamide was also formed, a second step was introduced where the crude product above was treated with a 1:1 mixture of 1,4-dioxane and 2 M NaOH. The crude sulphonamide resulting from subsequent acidification of the reaction mixture was isolated by filtration, washed well with water, and dried. Purification was carried out by flash column chromatography (2% MeOH/CH_2_Cl_2_ as eluant), giving the title compound as a pale yellow solid (211 mg, 54%), mp (MeOH/CH_2_Cl_2_) 266-269^o^C. ^1^H NMR [400 MHz, (CD_3_)_2_SO] δ 11.15 (br s, 1 H), 8.69 (d, *J* = 2.0 Hz, 1 H), 8.25 (d, *J* = 2.4 Hz, 1 H), 8.01 (dt, *J* = 8.7, 6.3 Hz, 1 H), 7.95 (s, 1 H), 7.83 (dd, *J* = 8.0, 1.5 Hz, 1 H), 7.68-7.75 (m, 3 H), 7.65 (d, *J* = 3.9 Hz, 1 H), 7.58 (dt, *J* = 8.9, 2.4 Hz, 1 H), 7.30 (dt, *J* = 8.2, 2.0 Hz, 1 H), 4.52 (s, 2 H), 3.09 (s, 3 H).

**General procedure D: 2,4-Difluoro-*N*-(5-(5-(2-methyl-1-oxoisoindolin-5-yl)thiophen-2-yl)pyridin-3-yl)benzenesulfonamide, sodium salt (5.Na).** 2,4-Difluoro-*N*-(5-(5-(2-methyl-1-oxoisoindolin-5-yl)thiophen-2-yl)pyridin-3-yl)benzenesulfonamide (**5**) (940 mg, 1.89 mmol) was suspended in EtOH (100 mL), then an aqueous solution of 1 M NaOH (1.89 mL) added. After stirring for 2 h. at room temperature, the solution was filtered through a pad of celite to remove minor insoluble impurities, washing well with EtOH. The resulting combined filtrates were combined and concentrated to ca 20 mL, then diluted with an equal volume of Et_2_O which resulted in crystallization of the title compound as a pale yellow solid (919 mg, 94%). ^1^H NMR [400 MHz, (CD_3_)_2_SO] δ 8.07 (d, *J* = 2.1 Hz, 1 H), 7.91 (m, 2 H), 7.78-7.88 (m, 2 H), 7.68 (d, *J* = 7.9 Hz, 1 H), 7.65 (d, *J* = 3.8 Hz, 1 H), 7.40-7.43 (m, 2 H), 7.18 (ddd, *J* = 9.7, 2.5 Hz, 1 H), 7.04-7.11 (m, 1 H), 4.51 (s, 2 H), 3.08 (s, 3 H).

**5-(Thiophen-2-yl)isoindolin-1-one (24).** 5-Bromoisoindolin-1-one and thiophene-2-boronic acid were reacted according to general procedure A. The title compound was isolated as a glossy cream solid (63%). ^1^H NMR [400 MHz, (CD_3_)_2_SO] δ 8.51 (br s, 1 H), 7.77 (dd, *J* = 7.9, 1.5 Hz, 1 H), 7.68 (d, *J* = 8.0 Hz, 1 H), 7.61-7.65 (m, 3 H), 7.19 (dd, *J* = 5.1, 3.7 Hz, 1 H), 4.41 (s, 2 H). LRMS (APCI^+^) calcd for C_12_H_10_NOS 216 (MH^+^), found 216.

**5-(5-Iodothiophen-2-yl)isoindolin-1-one (36).** Iodination of 5-(thiophen-2-yl)isoindolin-1-one (**24**) with *N*-iodosuccinimide according to general procedure B gave the title compound as a fluffy cream solid (84%). ^1^H NMR [400 MHz, (CD_3_)_2_SO] δ 8.54 (br s, 1 H), 7.80 (s, 1 H), 7.72 (dd, *J* = 7.9, 1.3 Hz, 1 H), 7.67 (d, *J* = 7.9 Hz, 1 H), 7.41 (d, *J* = 3.8 Hz, 1 H), 7.36 (d, *J* = 3.8 Hz, 1 H), 4.40 (s, 2 H). LRMS (APCI^+^) calcd for C_12_H_9_INOS 342 (MH^+^), found 342.

***tert*-Butyl (5-(5-(1-oxoisoindolin-5-yl)thiophen-2-yl)pyridin-3-yl)carbamate (55)**. 5-(5-Iodothiophen-2-yl)isoindolin-1-one (**36**) was reacted with *tert-*butyl (5-(4,4,5,5-tetramethyl-1,3,2-dioxaborolan-2-yl)pyridin-3-yl)carbamate according to general procedure A to give the title compound as a pale yellow solid (93%). ^1^H NMR [400 MHz, (CD_3_)_2_SO] δ 9.75 (br s, 1 H), 8.60 (d, *J* = 2.1 Hz, 1 H), 8.59 (br s, 1 H), 8.25 (br s, 1 H), 7.93 (br s, 1 H), 7.84 (dd, *J* = 7.9, 1.4 Hz, 1 H), 7.69-7.74 (m, 3 H), 7.65 (d, *J* = 3.9 Hz, 1 H), 4.43 (s, 2 H), 1.51 (s, 9 H). LRMS (APCI^+^) calcd for C_22_H_22_N_3_O_3_S 408 (MH^+^), found 408.

**General procedure E: 5-(5-(5-Aminopyridin-3-yl)thiophen-2-yl)isoindolin-1-one (63).** *tert*-Butyl (5-(5-(1-oxoisoindolin-5-yl)thiophen-2-yl)pyridin-3-yl)carbamate (**55**) (555 mg, 1.36 mmol) was dissolved in a mixture of CH_2_Cl_2_ (5 mL) and trifluoroacetic acid (5 mL) and stirred at room temperature for 1 h. The solvent was removed under reduced pressure, then the resulting solid suspended in acetone (12 mL) and stirred until homogenous. An equal volume of sat. NaHCO_3_ was carefully added and the resulting suspension collected by filtration, washed well with water and dried under vacuum. The title compound was isolated as a pale green solid (408 mg, 97%). ^1^H NMR [400 MHz, (CD_3_)_2_SO] δ 8.57 (br s, 1 H), 8.12 (d, *J* = 2.0 Hz, 1 H), 7.88-7.92 (m, 2 H), 7.84 (dd, *J* = 8.0, 1.5 Hz, 1 H), 7.72 (d, *J* = 8.2 Hz, 1 H), 7.69 (d, *J* = 3.8 Hz, 1 H), 7.54 (d, *J* = 3.8 Hz, 1 H), 7.16 (t, *J* = 2.3 Hz, 1 H), 5.51 (br s, 2 H), 4.43 (s, 2 H). LRMS (APCI^+^) calcd for C_17_H_14_N_3_OS 308 (MH^+^), found 308.

**2,4-Difluoro-*N*-(5-(5-(1-oxoisoindolin-5-yl)thiophen-2-yl)pyridin-3-yl)benzenesulfonamide (6).** 5-(5-(5-Aminopyridin-3-yl)thiophen-2-yl)isoindolin-1-one (**63**) was reacted with 2,4-difluorobenzenesulfonyl chloride according to general procedure C to give the title compound as a pale pink-orange solid (21%), mp (MeOH/CH_2_Cl_2_) 298-302^o^C. ^1^H NMR [400 MHz, (CD_3_)_2_SO] δ 11.51 (br s, 1 H), 8.69 (d, *J* = 2.0 Hz, 1 H), 8.60 (br s, 1 H), 8.25 (d, *J* = 2.4 Hz, 1 H), 7.97-8.05 (m, 1 H), 7.93 (br s, 1 H), 7.84 (dd, *J* = 7.9, 1.5 Hz, 1 H), 7.69-7.74 (m, 3 H), 7.65 (d, *J* = 3.9 Hz, 1 H), 7.54-7.61 (m, 1 H), 7.28-7.34 (m, 1 H), 4.43 (s, 2 H). LRMS (APCI^+^) calcd for C_23_H_16_F_2_N_3_O_3_S_2_ 484 (MH^+^), found 484.

**6-(Thiophen-2-yl)isoindolin-1-one (25).** Reaction of 6-bromoisoindolin-1-one and thiophene-2-boronic acid according to general procedure A gave the title compound as a fluffy white solid (93%). ^1^H NMR [400 MHz, (CD_3_)_2_SO] δ 8.63 (br s, 1 H), 7.84 (dd, *J* = 7.9, 1.8 Hz, 1 H), 7.82 (br s, 1 H), 7.60-7.64 (m, 2 H), 7.54 (dd, *J* = 5.1, 1.1 Hz, 1 H), 7.16 (dd, *J* = 5.1, 3.6 Hz, 1 H), 4.40 (s, 2 H). LRMS (APCI^+^) calcd for C_12_H_10_NOS 216 (MH^+^), found 216.

**6-(5-Iodothiophen-2-yl)isoindolin-1-one (37).** Iodination of 6-(thiophen-2-yl)isoindolin-1-one (**25**) with *N*-iodosuccinimide according to general procedure B gave the title compound as a pale grey solid (83%). ^1^H NMR [400 MHz, (CD_3_)_2_SO] δ 8.64 (br s, 1 H), 7.78-7.85 (m, 2 H), 7.61 (dd, *J* = 7.7, 0.7 Hz, 1 H), 7.38 (d, *J* = 3.8 Hz, 1 H), 7.36 (d, *J* = 3.8 Hz, 1 H), 4.39 (s, 2 H). LRMS (APCI^+^) calcd for C_12_H_9_INOS 342 (MH^+^), found 342.

***tert-*Butyl (5-(5-(3-oxoisoindolin-5-yl)thiophen-2-yl)pyridin-3-yl)carbamate (56).** 6-(5-Iodothiophen-2-yl)isoindolin-1-one (**37**) was reacted with *tert-*butyl (5-(4,4,5,5-tetramethyl-1,3,2-dioxaborolan-2-yl)pyridin-3-yl)carbamate according to general procedure A to give the title compound as a pale yellow crystalline solid (89%). ^1^H NMR [400 MHz, (CD_3_)_2_SO] δ 9.73 (br s, 1 H), 8.66 (br s 1 H), 8.60 (d, *J* = 2.1 Hz, 1 H), 8.51 (d, *J* = 2.2 Hz, 1 H), 8.22 (br s, 1 H), 7.92-7.97 (m, 2 H), 7.72 (d, *J* = 3.8 Hz, 1 H), 7.64 (dd, *J* = 7.8, 0.5 Hz, 1 H), 7.62 (d, *J* = 3.8 Hz, 1 H), 4.42 (s, 2 H), 1.51 (s, 9 H). LRMS (APCI^+^) calcd for C_22_H_22_N_3_O_3_S 408 (MH^+^), found 408.

**6-(5-(5-Aminopyridin-3-yl)thiophen-2-yl)isoindolin-1-one (64).** Deprotection of *tert-*butyl (5-(5-(3-oxoisoindolin-5-yl)thiophen-2-yl)pyridin-3-yl)carbamate (**56**) according to general procedure E gave the title compound as an off-white solid (98%). ^1^H NMR [400 MHz, (CD_3_)_2_SO] δ 8.65 (s, 1 H), 8.12 (d, *J* = 2.0 Hz, 1 H), 7.90-7.95 (m, 2 H), 7.89 (d, *J* = 2.5 Hz, 1 H), 7.67 (d, *J* = 3.8 Hz, 1 H), 7.64 (dd, *J* = 7.8, 0.5 Hz, 1 H), 7.51 (d, *J* = 3.8 Hz, 1 H), 7.16 (t, *J* = 2.3 Hz, 1 H), 5.49 (s, 2 H), 4.41 (s, 2 H). LRMS (APCI^+^) calcd for C_17_H_14_N_3_OS 308 (MH^+^), found 308.

**2,4-Difluoro-*N*-(5-(5-(3-oxoisoindolin-5-yl)thiophen-2-yl)pyridin-3-yl)benzenesulfonamide (7).** 6-(5-(5-Aminopyridin-3-yl)thiophen-2-yl)isoindolin-1-one (**64**) was reacted with 2,4-difluorobenzenesulfonyl chloride according to general procedure C to give the title compound as a pale yellow solid (65%), mp (MeOH/CH_2_Cl_2_) >300^o^C. ^1^H NMR [400 MHz, (CD_3_)_2_SO] δ 11.14 (br s, 1 H), 8.66 (br s, 2 H), 8.24 (d, *J* = 2.3 Hz, 1 H), 7.98-8.05 (m, 1 H), 7.92-8.04 (m, 2 H), 7.70-7.73 (m, 2 H), 7.65 (dd, *J* = 7.7, 0.7 Hz, 1 H), 7.62 (3.8 Hz, 1 H), 7.53-7.59 (m, 1 H), 7.27-7.33 (m, 1 H), 4.42 (s, 2 H). LRMS (APCI^+^) calcd for C_23_H_16_F_2_N_3_O_3_S_2_ 484 (MH^+^), found 484.

**2-Methyl-6-(thiophen-2-yl)isoindolin-1-one (26).** 6-Bromo-2-methylisoindolin-1-one was reacted with thiophene-2-boronic acid according to general procedure A to give the title compound as an off-white solid (97%). ^1^H NMR [400 MHz, CDCl_3_] δ 8.08 (d, *J* = 1.4 Hz, 1 H), 7.77 (dd, *J* = 7.9, 1.8 Hz, 1 H), 7.43 (dd, *J* = 7.9, 0.7 Hz, 1 H), 7.40 (dd, *J* = 3.6, 1.2 Hz, 1 H), 7.31 (dd, *J* = 5.1, 1.1 Hz, 1 H), 7.10 (dd, *J* = 5.1, 3.6 Hz, 1 H), 4.40 (s, 2 H), 3.22 (s, 3 H). LRMS (APCI^+^) calcd for C_13_H_12_NOS 230 (MH^+^), found 230.

**6-(5-Iodothiophen-2-yl)-2-methylisoindolin-1-one (38).** Iodination of 2-methyl-6-(thiophen-2-yl)isoindolin-1-one (**26**) with *N*-iodosuccinimide according to general procedure B gave the title compound as a pale brown crystalline solid (88%). ^1^H NMR [400 MHz, CDCl_3_] δ 7.99 (d, *J* = 1.4 Hz, 1 H), 7.66 (dd, *J* = 7.9, 1.8 Hz, 1 H), 7.43 (dd, *J* = 7.9, 0.7 Hz, 1 H), 7.74 (d, *J* = 3.8 Hz, 1 H), 7.05 (d, *J* = 3.8 Hz, 1 H), 4.39 (s, 2 H), 3.22 (s, 3 H). LRMS (APCI^+^) calcd for C_13_H_11_INOS 356 (MH^+^), found 356.

***tert-*Butyl (5-(5-(2-methyl-3-oxoisoindolin-5-yl)thiophen-2-yl)pyridin-3-yl)carbamate (57).** 6-(5-Iodothiophen-2-yl)-2-methylisoindolin-1-one (**38**) was reacted with *tert-*butyl (5-(4,4,5,5-tetramethyl-1,3,2-dioxaborolan-2-yl)pyridin-3-yl)carbamate according to general procedure A to give the title compound as a pale yellow solid (76%). ^1^H NMR [400 MHz, (CD_3_)_2_SO] δ 9.73 (br s, 1 H), 8.60 (d, *J* = 2.0 Hz, 1 H), 8.51 (d, *J* = 2.2 Hz, 1 H), 8.23 (br s, 1 H), 7.91-7.96 (m, 2 H), 7.73 (d, *J* = 3.9 Hz, 1 H), 7.65 (d, *J* = 8.5 Hz, 1 H), 7.61 (d, *J* = 3.8 Hz, 1 H), 4.50 (s, 2 H), 3.10 (s, 3 H), 1.51 (s, 9 H). LRMS (APCI^+^) calcd for C_23_H_24_N_3_O_3_S 422 (MH^+^), found 422.

**6-(5-(5-Aminopyridin-3-yl)thiophen-2-yl)-2-methylisoindolin-1-one (65).** Deprotection of *tert-*butyl (5-(5-(2-methyl-3-oxoisoindolin-5-yl)thiophen-2-yl)pyridin-3-yl)carbamate (**57**) according to general procedure E gave the title compound as a cream solid (100%). ^1^H NMR [400 MHz, (CD_3_)_2_SO] δ 8.12 (d, *J* = 2.0 Hz, 1 H), 7.86-7.92 (m, 3 H), 7.68 (d, *J* = 3.8 Hz, 1 H), 7.64 (d, *J* = 8.4 Hz, 1 H), 7.51 (d, *J* = 3.8 Hz, 1 H), 7.16 (t, *J* = 2.3 Hz, 1 H), 5.49 (br s, 2 H), 4.50 (s, 2 H), 3.10 (s, 3 H). LRMS (APCI^+^) calcd for C_18_H_16_N_3_OS 322 (MH^+^), found 322.

**2,4-Difluoro-*N*-(5-(5-(2-methyl-3-oxoisoindolin-5-yl)thiophen-2-yl)pyridin-3-yl)benzenesulfonamide (8).** 6-(5-(5-Aminopyridin-3-yl)thiophen-2-yl)-2-methylisoindolin-1-one (**65**) was reacted with 2,4-difluorobenzenesulfonyl chloride according to general procedure C to give the title compound as an off-white solid (47%), mp (MeOH/CH_2_Cl_2_) 292-295^o^C. ^1^H NMR [400 MHz, (CD_3_)_2_SO] δ 11.14 (br s, 1 H), 8.68 (d, *J* = 1.9 Hz, 1 H), 8.24 (d, *J* = 2.3 Hz, 1 H), 7.98-8.05 (m, 1 H), 7.91-7.95 (m, 2 H), 7.71-7.75 (m, 2 H), 7.66 (d, *J* = 8.5 Hz, 1 H), 7.62 (d, *J* = 3.8 Hz, 1 H), 7.53-7.60 (m, 1 H), 7.26-7.32 (m, 1 H), 4.51 (s, 2 H), 3.10 (s, 3 H). LRMS (APCI^+^) calcd for C_24_H_18_F_2_N_3_O_3_S_2_ 498 (MH^+^), found 498.

**6-(Thiophen-2-yl)-3,4-dihydroisoquinolin-1(2*H*)-one (27).**  6-Bromo-3,4-dihydroisoquinolin-1(2*H*)-one was reacted with thiophene-2-boronic acid according to general procedure A to give the title compound as an off-white solid (100%). ^1^H NMR [400 MHz, (CD_3_)_2_SO] δ 7.92 (br s, 1 H), 7.85 (d, *J* = 8.0 Hz, 1 H), 7.59-7.65 (m, 4 H), 7.17 (t, *J* = 4.3 Hz, 1 H), 3.36-3.41 (m, 2 H), 2.95 (t, *J* = 6.6 Hz, 2 H). LRMS (APCI^+^) calcd for C_13_H_12_NOS 230 (MH^+^), found 230.

**6-(5-(5-Aminopyridin-3-yl)thiophen-2-yl)-3,4-dihydroisoquinolin-1(2*H*)-one (39).** 6-(Thiophen-2-yl)-3,4-dihydroisoquinolin-1(2*H*)-one (**27**) was iodinated according to general procedure B to give 6-(5-iodothiophen-2-yl)-3,4-dihydroisoquinolin-1(2*H*)-one (**50**) which was then used directly in a reaction with 5-(4,4,5,5-tetramethyl-1,3,2-dioxaborolan-2-yl)pyridin-3-amine according to general procedure A. After refluxing for 16 h., the solution was allowed to cool to room temperature and extracted into 5% MeOH/CH_2_Cl_2_ and washed with H_2_O. The aqueous layer was further extracted with 5% MeOH/CH_2_Cl_2_ and the organic extracts dried (Na_2_SO_4_), filtered, and concentrated to give a crude solid which was dried onto silica gel. The crude material was chromatographed (1-5% MeOH/CH_2_Cl_2_) to give the title compound as a green solid (86%); mp (CH_2_Cl_2_/MeOH) 277-280^o^C. ^1^H NMR [400 MHz, (CD_3_)_2_SO] δ 8.12 (d, *J* = 2.0 Hz, 1 H), 7.94 (br s, 1 H), 7.89 (d, *J* = 2.5 Hz, 1 H), 7.87 (d, *J* = 8.0 Hz, 1 H), 7.61 (m, 3 H), 7.53 (d, *J* = 3.8 Hz, 1 H), 7.15 (t, *J* = 2.2 Hz, 1 H), 5.50 (s, 2 H), 3.40 (dt, *J* = 6.6, 2.7 Hz, 2 H), 2.96 (t, *J* = 6.5 Hz, 2 H).

**2,4-Difluoro-*N*-(5-(5-(1-oxo-1,2,3,4-tetrahydroisoquinolin-6-yl)thiophen-2-yl)pyridin-3-yl)benzensulphonamide (9).**  6-(5-(5-Aminopyridin-3-yl)thiophen-2-yl)-3,4-dihydroisoquinolin-1(2*H*)-one (**50**) was reacted with 2,4-difluorobenzenesulphonyl chloride according to general procedure C to give the title compound as a yellow solid (15%); mp 267-270^o^C. ^1^H NMR [400 MHz, (CD_3_)_2_SO] δ 11.14 (br s, 1 H), 8.69 (d, *J* = 1.6 Hz, 1 H), 8.25 (d, *J* = 2.2 Hz, 1 H), 7.96-8.06 (m, 1 H), 7.95 (br s, 1 H), 7.88 (d, *J* = 8.0 Hz, 1 H), 7.65-7.76 (m, 4 H), 7.64 (d, *J* = 3.8 Hz, 1 H), 7.57 (dt, *J* = 8.4, 2.3 Hz, 1 H), 7.30 (dt, *J* = 8.4, 2.3 Hz, 1 H), 3.40 (dt, *J* = 6.6, 2.4 Hz, 2 H), 2.97 (t, *J* = 6.4 Hz, 2 H).

**General procedure F: 2-Methyl-6-(thiophen-2-yl)-3,4-dihydroisoquinolin-1(2*H*)-one (28).** 6-(Thiophen-2-yl)-3,4-dihydroisoquinolin-1(2*H*)-one (**27**, 1.63 g, 7.12 mmol) was dissolved in dry DMF (50 mL) and cooled to 0^o^C. NaH (313 mg, 7.82 mmol) was added and the mixture stirred for 0.5 h., gradually being allowed to return to room temperature. Methyl iodide (1.11 g, 7.82 mmol) was then added dropwise and stirring continued for 1.5 h. The mixture was concentrated under reduced pressure (high vacuum) and the resulting oil dissolved in EtOAc (100 mL) which was washed with water (2x100 mL), brine (100 mL) and dried over Na_2_SO_4_. Filtration and concentration gave a crystalline solid which was purified by chromatography on silica gel (0-2% acetone/CH_2_Cl_2_) to give the title compound as a cream solid (1.35 g, 78%). ^1^H NMR [400 MHz, (CD_3_)_2_SO] δ 7.87 (d, *J* = 8.1 Hz, 1 H), 7.61-7.65 (m, 3 H), 5.57 (br s, 1 H), 7.17 (dd, *J* = 4.9, 3.8 Hz, 1 H), 3.56 (t, *J* = 6.7 Hz, 2 H), 3.00-3.04 (m, 5 H). LRMS (APCI^+^) calcd for C_14_H_14_NOS 244 (MH^+^), found 244.

**6-(5-Iodothiophen-2-yl)-2-methyl-3,4-dihydroisoquinolin-1(2*H*)-one (40).** Iodination of 2-methyl-6-(thiophen-2-yl)-3,4-dihydroisoquinolin-1(2*H*)-one (**28**) with *N*-iodosuccinimide according to general procedure B gave the title compound as a cream solid (37%). ^1^H NMR [400 MHz, (CD_3_)_2_SO] δ 7.86 (d, *J* = 8.0 Hz, 1 H), 7.52-7.60 (m, 2 H), 7.40 (d, *J* = 3.8 Hz, 1 H), 7.34 (d, *J* = 3.8 Hz, 1 H), 3.55 (t, *J* = 6.7 Hz, 2 H), 2.97-3.04 (m, 5 H). LRMS (APCI^+^) calcd for C_14_H_13_INOS 370 (MH^+^), found 370.

***tert-*Butyl (5-(5-(2-methyl-1-oxo-1,2,3,4-tetrahydroisoquinolin-6-yl)thiophen-2-yl)pyridin-3-yl)carbamate (58).** 6-(5-Iodothiophen-2-yl)-2-methyl-3,4-dihydroisoquinolin-1(2*H*)-one (**40**) was reacted with *tert-*butyl (5-(4,4,5,5-tetramethyl-1,3,2-dioxaborolan-2-yl)pyridin-3-yl)carbamate according to general procedure A to give the title compound as a cream solid (55%). ^1^H NMR [400 MHz, (CD_3_)_2_SO] δ 9.74 (br s, 1 H), 8.61 (d, *J* = 2.1 Hz, 1 H), 8.50 (d, *J* = 2.3 Hz, 1 H), 8.26 (br s, 1 H), 7.90 (d, *J* = 8.1 Hz, 1 H), 7.68-7.72 (m, 2 H), 7.63-7.67 (m, 2 H), 3.57 (t, *J* = 6.7 Hz, 2 H), 3.02-3.07 (m, 5 H), 1.51 (s, 9 H). LRMS (APCI^+^) calcd for C_24_H_26_N_3_O_3_S 436 (MH^+^), found 436.

**6-(5-(5-Aminopyridin-3-yl)thiophen-2-yl)-2-methyl-3,4-dihydroisoquinolin-1(2*H*)-one (66).** Deprotection of *tert-*butyl (5-(5-(2-methyl-1-oxo-1,2,3,4-tetrahydroisoquinolin-6-yl)thiophen-2-yl)pyridin-3-yl)carbamate (**58**) according to general procedure E gave the title compound as a cream solid (92%). ^1^H NMR [400 MHz, (CD_3_)_2_SO] δ 8.12 (d, *J* = 2.0 Hz, 1 H), 7.87-7.91 (m, 2 H), 7.64-7.68 (m, 2 H), 7.62 (br s, 1 H), 7.53 (d, *J* = 3.8 Hz, 1 H), 7.16 (t, *J* = 2.3 Hz, 1 H), 3.57 (t, *J* = 6.7 Hz, 2 H), 3.01-3.06 (m, 5 H). LRMS (APCI^+^) calcd for C_19_H_18_N_3_OS 336 (MH^+^), found 336.

**2,4-Difluoro-*N*-(5-(5-(2-methyl-1-oxo-1,2,3,4-tetrahydroisoquinolin-6-yl)thiophen-2-yl)pyridin-3-yl)benzenesulfonamide (10).** (5-(5-Aminopyridin-3-yl)thiophen-2-yl)-2-methyl-3,4-dihydroisoquinolin-1(2*H*)-one (**66**) was reacted with 2,4-difluorobenzenesulfonyl chloride according to general procedure C to give the title compound as an off-white solid (93%), mp (MeOH/CH_2_Cl_2_) 262-265^o^C. ^1^H NMR [400 MHz, (CD_3_)_2_SO] δ 11.15 (br s, 1 H), 8.67 (br s, 1 H), 8.24 (d, *J* = 1.9 Hz, 1 H), 7.96-8.03 (m, 1 H), 7.91 (d, *J* = 8.1 Hz, 1 H), 7.62-7.73 (m, 5 H), 7.52-7.59 (m, 1 H), 7.26-7.33 (m, 1 H), 3.57 (t, *J* = 6.7 Hz, 2 H), 3.01-3.07 (m, 5 H). LRMS (APCI^+^) calcd for C_25_H_20_F_2_N_3_O_3_S_2_ 512 (MH^+^), found 512.

**Methyl 4-bromophenethylcarbamate.** Adaption of a literature procedure^1^ gave the title compound as a pale yellow oil (94%). ^1^H NMR [400 MHz, CDCl_3_] δ 7.43 (d, *J* = 8.4 Hz, 2 H), 7.06 (d, *J* = 8.3 Hz, 2 H), 4.65 (bs, 1 H), 3.65 (s, 3 H), 3.41 (q, *J* = 6.3 Hz, 2 H), 2.77 (t, *J* = 7.0 Hz, 2 H). LRMS (APCI^+^) calcd for C_10_H_13_BrNO_2_ 259 (MH^+^), found 259.

**7-Bromo-3,4-dihydroisoquinolin-1(2*H*)-one.** Cyclisation of methyl 4-bromophenethylcarbamate was carried out by adaption of a literature procedure^2^ to give the title compound as a white solid (20%). ^1^H NMR [400 MHz, CDCl_3_] δ 8.20 (d, *J* = 2.1 Hz, 1 H), 7.55 (dd, *J* = 8.1, 2.2 Hz, 1 H), 7.09 (d, *J* = 8.1 Hz, 1 H), 6.12 (bs, 1 H), 3.55 (dt, *J* = 6.2, 2.9 Hz, 2 H), 2.94 (t, *J* = 6.6 Hz, 2 H). LRMS (APCI^+^) calcd for C_9_H_9_BrNO 227 (MH^+^), found 227.

**General procedure G: 7-(4,4,5,5-Tetramethyl-1,3,2-dioxaborolan-2-yl)-3,4-dihydroisoquinolin-1(2*H*)-one.** 7-Bromo-3,4-dihydroisoquinolin-1(2*H*)-one (670 mg, 2.96 mmol), bis(pinacolato)diboron (825 mg, 3.26 mmol), KOAc (872 mg, 8.89 mmol) and Pd(dppf)Cl_2_ (121 mg, 0.15 mmol) were weighed into a flask, DMSO (15 mL) added, and the entire mixture heated and stirred under N_2_ for 4 h. Upon cooling, the reaction was diluted with CH_2_Cl_2_ (100 mL) and filtered through a pad of celite, washing well with additional CH_2_Cl_2_. The filtrate and combined washings (ca 200 mL) was washed with water (3x100 mL), brine (100 mL), dried (Na_2_SO_4_) and filtered. Removal of the solvent under reduced pressure gave a black-brown oil which was purified by flash column chromatography on silica gel (50% EtOAc/hexanes as eluant). The title compound was isolated as a light brown solid (378 mg, 47%). ^1^H NMR [400 MHz, CDCl_3_] δ 8.53 (s, 1 H), 7.86 (dd, *J* = 7.5, 1.3 Hz, 1 H), 7.21 (d, *J* = 7.4 Hz, 1 H), 5.88 (br s, 1 H), 3.55 (dt, *J* = 6.6, 2.9 Hz, 2 H), 3.01 (t, *J* = 6.6 Hz, 2 H), 1.33 (s, 12 H). LRMS (APCI^+^) calcd for C_15_H_21_BNO_3_ 274 (MH^+^), found 274.

**7-(Thiophen-2-yl)-3,4-dihydroisoquinolin-1(2*H*)-one (29).** 7-(4,4,5,5-Tetramethyl-1,3,2-dioxaborolan-2-yl)-3,4-dihydroisoquinolin-1(2*H*)-one was reacted with 2-bromothiophene according to general procedure A to give the title compound as an off-white solid (74%). ^1^H NMR [400 MHz, CDCl_3_] δ 8.33 (d, *J* = 2.0 Hz, 1 H), 7.69 (dd, *J* = 7.9 Hz, 1 H), 7.38 (dd, *J* = 3.6, 1.1 Hz, 1 H), 7.29 (dd, *J* = 5.1, 1.1 Hz, 1 H), 7.24 (d, *J* = 8.0 Hz, 1 H), 7.09 (dd, *J* = 5.1, 3.6 Hz, 1 H), 6.02 (br s, 1 H), 3.56-3.62 (m, 2 H), 3.02 (t, *J* = 6.6 Hz, 2 H). LRMS (APCI^+^) calcd for C_13_H_12_NOS 230 (MH^+^), found 230.

**7-(5-Iodothiophen-2-yl)-3,4-dihydroisoquinolin-1(2*H*)-one (41).** Iodination of 7-(thiophen-2-yl)-3,4-dihydroisoquinolin-1(2*H*)-one (**29**) with *N*-iodosuccinimide according to general procedure B gave the title compound as a pale grey solid (79%). ^1^H NMR [400 MHz, CDCl_3_] δ 8.04 (br s, 1 H), 7.97 (d, *J* = 2.0 Hz, 1 H), 7.72 (dd, *J* = 7.9, 2.1 Hz, 1 H), 7.34-7.38 (m, 2 H), 7.27 (d, *J* = 3.8 Hz, 1 H), 3.35-3.40 (m, 2 H), 2.91 (t, *J* = 6.6 Hz, 2 H). LRMS (APCI^+^) calcd for C_13_H_11_INOS 356 (MH^+^), found 356.

***tert*-Butyl (5-(5-(1-oxo-1,2,3,4-tetrahydroisoquinolin-7-yl)thiophen-2-yl)pyridin-3-yl)carbamate (59).** 7-(5-Iodothiophen-2-yl)-3,4-dihydroisoquinolin-1(2*H*)-one (**41**) was reacted with *tert-*butyl (5-(4,4,5,5-tetramethyl-1,3,2-dioxaborolan-2-yl)pyridin-3-yl)carbamate according to general procedure A to give the title compound as a dark yellow solid (88%). ^1^H NMR [400 MHz, (CD_3_)_2_SO] δ 9.72 (br s, 1 H), 8.59 (d, *J* = 2.0 Hz, 1 H), 8.51 (d, *J* = 2.3 Hz, 1 H), 8.23 (br s, 1 H), 8.10 (d, *J* = 2.0 Hz, 1 H), 8.06 (br s, 1 H), 7.85 (dd, *J* = 7.9, 2.1 Hz, 1 H), 7.62 (d, *J* = 3.8 Hz, 1 H), 7.60 (d, *J* = 3.8 Hz, 1 H), 7.40 (d, *J* = 8.0 Hz, 1 H), 3.37-3.42 (m, 2 H), 2.93 (t, *J* = 6.5 Hz, 2 H), 1.51 (s, 9 H). LRMS (APCI^+^) calcd for C_23_H_24_N_3_O_3_S 422 (MH^+^), found 422.

**7-(5-(5-Aminopyridin-3-yl)thiophen-2-yl)-3,4-dihydroisoquinolin-1(2*H*)-one (67).** Deprotection of *tert*-butyl (5-(5-(1-oxo-1,2,3,4-tetrahydroisoquinolin-7-yl)thiophen-2-yl)pyridin-3-yl)carbamate (**59**) according to general procedure E gave the title compound as a pale yellow solid (98%). ^1^H NMR [400 MHz, (CD_3_)_2_SO] δ 8.12 (d, *J* = 2.0 Hz, 1 H), 8.09 (d, *J* = 2.0 Hz, 1 H), 8.05 (br s, 1 H), 7.89 (d, *J* = 2.4 Hz, 1 H), 7.83 (dd, *J* = 7.9, 2.1 Hz, 1 H), 7.59 (d, *J* = 3.8 Hz, 1 H), 7.50 (d, *J* = 3.9 Hz, 1 H), 7.40 (d, *J* = 8.0 Hz, 1 H), 7.18 (t, *J* = 2.2 Hz, 1 H), 5.49 (br s, 2 H), 3.37-3.43 (m, 2 H), 2.94 (t, *J* = 6.5 Hz, 2 H). LRMS (APCI^+^) calcd for C_18_H_16_N_3_OS 322 (MH^+^), found 322.

**2,4-Difluoro-*N*-(5-(5-(1-oxo-1,2,3,4-tetrahydroisoquinolin-7-yl)thiophen-2-yl)pyridin-3-yl)benzenesulfonamide (11).** 7-(5-(5-Aminopyridin-3-yl)thiophen-2-yl)-3,4-dihydroisoquinolin-1(2*H*)-one (**67**) was reacted with 2,4-difluorobenzenesulfonyl chloride according to general procedure C to give the title compound as a dark yellow solid (74%), mp (MeOH/CH_2_Cl_2_) 252^o^C (dec.). ^1^H NMR [400 MHz, (CD_3_)_2_SO] δ 11.14 (br s, 1 H), 8.70 (d, *J* = 2.0 Hz, 1 H), 8.25 (d, *J* = 2.4 Hz, 1 H), 8.10 (d, *J* = 2.1 Hz, 1 H), 8.07 (br s, 1 H), 7.98-8.05 (m, 1 H), 7.84 (dd, *J* = 7.8, 2.1 Hz, 1 H), 7.74 (t, *J* = 2.2 Hz, 1 H), 7.54-7.64 (m, 3 H), 7.41 (d, *J* = 8.0 Hz, 1 H), 7.27-7.33 (m, 1 H), 3.37-3.43 (m, 2 H), 2.94 (t, *J* = 6.5 Hz, 2 H). LRMS (APCI^+^) calcd for C_24_H_18_F_2_N_3_O_3_S_2_ 498 (MH^+^), found 498.

**7-Bromo-2-methyl-3,4-dihydroisoquinolin-1(2*H*)-one.**  7-Bromo-3,4-dihydroisoquinolin-1(2*H*)-one was methylated according to general procedure F to give the title compound as a pale yellow waxy solid (95%). ^1^H NMR [400 MHz, CDCl_3_] δ 8.21 (d, *J* = 2.2 Hz, 1 H), 7.52 (dd, *J* = 8.0, 2.1 Hz, 1 H), 7.05 (d, *J* = 7.9 Hz, 1 H), 3.56 (t, *J* = 6.6 Hz, 2 H), 3.15 (s, 3 H), 2.96 (t, *J* = 6.8 Hz, 2 H). LRMS (APCI^+^) calcd for C_10_H_11_BrNO 240, 242 (MH^+^), found 240, 242.

**2-Methyl-7-(4,4,5,5-tetramethyl-1,3,2-dioxaborolan-2-yl)-3,4-dihydroisoquinolin-1(2*H*)-one.**  7-Bromo-2-methyl-3,4-dihydroisoquinolin-1(2*H*)-one was reacted with bis(pinacolato)diboron according to general procedure G to give the title compound as a white solid (100%). ^1^H NMR [400 MHz, CDCl_3_] δ 8.53 (d, *J* = 0.6 Hz, 7.82 (dd, *J* = 7.4, 1.2 Hz, 1 H), 7.16 (dd, *J* = 7.4, 0.4 Hz, 1 H), 3.55 (t, *J* = 6.7 Hz, 2 H), 3.15 (s, 3 H), 3.01 (t, *J* = 6.6 Hz, 2 H), 1.33 (s, 12 H). LRMS (APCI^+^) calcd for C_16_H_22_BNO_3_ 288 (MH^+^), found 288.

**2-Methyl-7-(thiophen-2-yl)-3,4-dihydroisoquinolin-1(2*H*)-one (30).** 2-Methyl-7-(4,4,5,5-tetramethyl-1,3,2-dioxaborolan-2-yl)-3,4-dihydroisoquinolin-1(2*H*)-one was reacted with 2-bromothiophene according to general procedure A to give the title compound as a cream solid (73%). ^1^H NMR [400 MHz, CDCl_3_] δ 8.34 (d, *J* = 2.0 Hz, 1 H), 7.64 (dd, *J* = 7.8, 2.1 Hz, 1 H), 7.38 (dd, *J* = 3.6, 1.2 Hz, 1 H), 7.28 (dd, *J* = 5.1, 1.1 Hz, 1 H), 7.18 (d, *J* = 7.7 Hz, 1 H), 7.08 (dd, *J* = 5.1, 3.6 Hz, 1 H), 3.58 (t, *J* = 6.7 Hz, 2 H), 3.18 (s, 3 H), 3.01 (t, *J* = 6.7 Hz, 2 H). LRMS (APCI^+^) calcd for C_14_H_14_NOS 244 (MH^+^), found 244.

**7-(5-Iodothiophen-2-yl)-2-methyl-3,4-dihydroisoquinolin-1(2*H*)-one (42).**  Iodination of 2-methyl-7-(thiophen-2-yl)-3,4-dihydroisoquinolin-1(2*H*)-one (**30**) with *N*-iodosuccinimide according to general procedure B gave the title compound as a waxy grey solid (93%). ^1^H NMR [400 MHz, (CD_3_)_2_SO] δ 7.99 (d, *J* = 2.0 Hz, 1 H), 7.72 (dd, *J* = 7.9, 2.1 Hz, 1 H), 7.37 (d, *J* = 3.8 Hz, 1 H), 7.34 (d, *J* = 8.0 Hz, 1 H), 7.26 (d, *J* = 3.8 Hz, 1 H), 3.56 (t, *J* = 6.7 Hz, 2 H), 3.04 (s, 3 H), 2.98 (t, *J* = 6.6 Hz, 2 H). LRMS (APCI^+^) calcd for C_14_H_13_INOS 370 (MH^+^), found 370.

***tert-*Butyl (5-(5-(2-methyl-1-oxo-1,2,3,4-tetrahydroisoquinolin-7-yl)thiophen-2-yl)pyridin-3-yl)carbamate (60).** 7-(5-Iodothiophen-2-yl)-2-methyl-3,4-dihydroisoquinolin-1(2*H*)-one (**42**) was reacted with *tert-*butyl (5-(4,4,5,5-tetramethyl-1,3,2-dioxaborolan-2-yl)pyridin-3-yl)carbamate according to general procedure A to give the title compound as a cream solid (92%). ^1^H NMR [400 MHz, (CD_3_)_2_SO] δ 9.72 (br s, 1 H), 8.59 (d, *J* = 2.0 Hz, 1 H), 8.51 (d, *J* = 2.3 Hz, 1 H), 8.22 (br s, 1 H), 8.12 (d, *J* = 2.0 Hz, 1 H), 7.84 (dd, *J* = 7.9, 2.1 Hz, 1 H), 7.59-7.63 (m, 2 H), 7.38 (d, *J* = 8.0 Hz, 1 H), 3.58 (t, *J* = 6.7 Hz, 2 H), 3.06 (s, 3 H), 3.01 (t, *J* = 6.6 Hz, 2 H), 1.51 (s, 9 H). LRMS (APCI^+^) calcd for C_24_H_26_N_3_O_3_S 436 (MH^+^), found 436.

**7-(5-(5-Aminopyridin-3-yl)thiophen-2-yl)-2-methyl-3,4-dihydroisoquinolin-1(2*H*)-one (68).** Deprotection of *tert-*butyl (5-(5-(2-methyl-1-oxo-1,2,3,4-tetrahydroisoquinolin-7-yl)thiophen-2-yl)pyridin-3-yl)carbamate (**60**) according to general procedure E gave the title compound as a pale yellow solid (100%). ^1^H NMR [400 MHz, (CD_3_)_2_SO] δ 8.12 (d, *J* = 2.0 Hz, 1 H), 8.10 (d, *J* = 2.0 Hz, 1 H), 7.88 (d, *J* = 2.5 Hz, 1 H), 7.81 (dd, *J* = 7.9, 2.1 Hz, 1 H), 7.57 (d, *J* = 3.8 Hz, 1 H), 7.50 (d, *J* = 3.8 Hz, 1 H), 7.37 (d, *J* = 8.0 Hz, 1 H), 7.17 (t, *J* = 2.3 Hz, 1 H), 3.57 (t, *J* = 6.7 Hz, 2 H), 3.05 (s, 3 H), 3.00 (t, *J* = 6.6 Hz, 2 H). LRMS (APCI^+^) calcd for C_19_H_18_N_3_OS 336 (MH^+^), found 336.

**2,4-Difluoro-*N*-(5-(5-(2-methyl-1-oxo-1,2,3,4-tetrahydroisoquinolin-7-yl)thiophen-2-yl)pyridin-3-yl)benzenesulfonamide (12).** 7-(5-(5-Aminopyridin-3-yl)thiophen-2-yl)-2-methyl-3,4-dihydroisoquinolin-1(2*H*)-one (**68**) was reacted with 2,4-difluorobenzenesulfonyl chloride according to general procedure C to give the title compound as an off-white solid (80%), mp (MeOH/CH_2_Cl_2_) 280-283^o^C. ^1^H NMR [400 MHz, (CD_3_)_2_SO] δ 11.12 (br s, 1 H), 8.67 (br s, 1 H), 8.24 (d, *J* = 2.4 Hz, 1 H), 8.11 (d, *J* = 2.0 Hz, 1 H), 7.97-8.04 (m, 1 H), 7.84 (dd, *J* = 7.8, 2.1 Hz, 1 H), 7.72 (t, *J* = 2.2 Hz, 1 H), 7.53-7.62 (m, 3 H), 7.37 (d, *J* = 8.0 Hz, 1 H), 7.26-7.32 (m, 1 H), 3.58 (t, *J* = 6.7 Hz, 2 H), 3.06 (s, 3 H), 3.01 (t, *J* = 6.7 Hz, 2 H). LRMS (APCI^+^) calcd for C_25_H_20_F_2_N_3_O_3_S_2_ 512 (MH^+^), found 512.

**5-Bromo-2,3-dimethylisoindolin-1-one.** Methyl 4-Bromo-2-ethylbenzoate (2.17 g, 8.93 mmol) (prepared according to a literature procedure^3^) was dissolved in benzene (24 mL), to which was added *N*-bromosuccinimide (1.75 g, 9.82 mmol) and azobisisobutyronitrile (147 mg, 0.89 mmol). This mixture was heated at 90^o^C overnight, allowed to cool, filtered and diluted with Et_2_O (150 mL). This solution was then washed with 1 M sodium metabisulfite (2x100 mL), water (100 mL) and brine (100 mL), dried (Na_2_SO_4_), filtered and the solvent removed under reduced pressure to afford the crude product as a pale yellow oil which was filtered through a plug of silica gel (5% Et_2_O/hexanes). The resulting transparent viscous oil (2.60 g, 90%) was then used directly in a subsequent cyclisation. This bromide (2.60 g, 8.07 mmol) was then dissolved in THF (30 mL), to which was added 33% MeNH_2_ in EtOH (20 mL) and the mixture stirred overnight. All solvent was removed under reduced pressure to give a residue which was purified by filtration through a plug of silica gel (50% EtOAc/hexanes), affording the title compound as a waxy white solid (1.80 g, 93%). ^1^H NMR [400 MHz, CDCl_3_] δ 7.69 (dd, *J* = 7.9, 0.5 Hz, 1 H), 7.56-7.60 (m, 2 H), 4.42 (q, *J* = 6.8 Hz, 1 H), 3.10 (s, 3 H), 1.13 (d, *J* = 6.8 Hz, 3 H). LRMS (APCI^+^) calcd for C_10_H_10_BrNO 241 (MH^+^), found 241.

**2,3-Dimethyl-5-(thiophen-2-yl)isoindolin-1-one (31).** 5-Bromo-2,3-dimethylisoindolin-1-one was reacted with thiophene-2-boronic acid according to general procedure A to give the title compound as a crystalline tan solid (100%). ^1^H NMR [400 MHz, (CD_3_)_2_SO] δ 7.92 (t, *J* = 0.9 Hz, 1 H), 7.76 (dd, *J* = 7.9, 1.3 Hz, 1 H), 7.65-7.68 (m, 2 H), 7.64 (dd, *J* = 5.1, 1.1 Hz, 1 H), 7.14 (dd, *J* = 5.1, 3.7 Hz, 1 H), 4.59 (q, *J* = 6.7 Hz, 1 H), 3.01 (s, 3 H), 1.47 (d, *J* = 6.8 Hz, 3 H). LRMS (APCI^+^) calcd for C_14_H_13_NOS 244 (MH^+^), found 244.

**5-(5-Iodothiophen-2-yl)-2,3-dimethylisoindolin-1-one (43).** Iodination of 2,3-dimethyl-5-(thiophen-2-yl)isoindolin-1-one (**31**) with *N*-iodosuccinimide according to general procedure B gave the title compound as a crystalline tan solid (94%). ^1^H NMR [400 MHz, (CD_3_)_2_SO] δ 7.88 (d, *J* = 0.7 Hz, 1 H), 7.70 (dd, *J* = 7.9, 1.4 Hz, 1 H), 7.65 (dd, *J* = 7.9, 0.3 Hz, 1 H), 7.42 (d, *J* = 3.8 Hz, 1 H), 7.40 (d, *J* = 3.8 Hz, 1 H), 4.58 (q, *J* = 3.7 Hz, 1 H), 3.01 (s, 3 H), 1.46 (d, *J* = 6.7 Hz, 3 H). LRMS (APCI^+^) calcd for C_14_H_12_INOS 370 (MH^+^), found 370.

**5-(5-(5-Aminopyridin-3-yl)thiophen-2-yl)-2,3-dimethylisoindolin-1-one (51).**  5-(5-iodothiophen-2-yl)-2,3-dimethylisoindolin-1-one (**43**) was reacted with 5-(4,4,5,5-tetramethyl-1,3,2-dioxaborolan-2-yl)pyridin-3-amine according to general procedure A. Purification was carried out by chromatography on silica gel (1-5% MeOH/CH_2_Cl_2_) to give the title compound as a light-brown solid (86%); mp (CH_2_Cl_2_/MeOH) 239-241^o^C. ^1^H NMR [400 MHz, (CD_3_)_2_SO] δ 8.13 (d, *J* = 2.0 Hz, 1 H), 7.97 (s, 1 H), 7.90 (d, *J* = 2.5 Hz, 1 H), 7.79 (dd, *J* = 7.9, 1.4 Hz, 1 H), 7.70 (t, *J* = 3.8 Hz, 1 H), 7.67 (s, 1 H), 7.54 (d, *J* = 3.8 Hz, 1 H), 7.17 (t, *J* = 2.3 Hz, 1 H), 5.50 (br s, 2 H), 4.60 (q, *J* = 6.6 Hz, 1 H), 3.02 (s, 3 H), 1.50 (d, *J* = 6.8 Hz, 3 H).

***N*-(5-(5-(2,3-Dimethyl-1-oxoisoindolin-5-yl)thiophen-2-yl)pyridin-3-yl)-2,4-difluorobenzenesulfonamide (13).** 5-(5-(5-Aminopyridin-3-yl)thiophen-2-yl)-2,3-dimethylisoindolin -1-one (**51**) was reacted with 2,4-difluorobenzenesulphonyl chloride according to general procedure C to give the title compound as a pink solid (19%); mp (CH_2_Cl_2_/MeOH) 229-230^o^C. ^1^H NMR [400 MHz, (CD_3_)_2_SO] δ 11.15 (br s, 1 H), 8.69 (d, *J* = 2.0 Hz, 1 H), 8.25 (d, *J* = 2.4 Hz, 1 H), 8.00 (m, 2 H), 7.82 (dd, *J* = 8.0, 1.4 Hz, 1 H), 7.74 (m, 2 H), 7.70 (d, *J* = 7.9 Hz, 1 H), 7.65 (d, *J* = 3.9 Hz, 1 H), 7.56 (dt, *J* = 8.2, 2.4 Hz, 1 H), 7.30 (dt, *J* = 8.2, 2.0 Hz, 1 H), 4.61 (q, *J* = 6.7 Hz, 1 H), 3.02 (s, 3 H), 1.50 (d, *J* = 0.2 Hz, 3 H).

**5-(Thiophen-2-yl)indolin-2-one (32).** 5-Bromooxindole was reacted with thiophene-2-boronic acid according to general procedure A to give the title compound as a pale brown crystalline solid (100%). ^1^H NMR [400 MHz, (CD_3_)_2_SO] δ 10.47 (s, 1 H), 7.51 (br s, 1 H), 7.46 (dd, *J* = 8.0, 1.9 Hz, 1 H), 7.44 (dd, *J* = 8.1, 1.1 Hz, 1 H), 7.36 (dd, *J* = 3.6, 1.2 Hz, 1 H), 7.09 (dd, *J* = 5.1, 3.6 Hz, 1 H), 6.84 (d, *J* = 8.0 Hz, 1 H), 3.53 (s, 2 H). LRMS (APCI^+^) calcd for C_12_H_9_NOS 216 (MH^+^), found 216.

**5-(5-Iodothiophen-2-yl)indolin-2-one (44)**. 5-(Thiophen-2-yl)indolin-2-one (**32**) was iodinated with *N*-iodosuccinimide according to general procedure B. The title compound was isolated as a pale brown solid (100%). ^1^H NMR [400 MHz, (CD_3_)_2_SO] δ 10.49 (br s, 1 H), 7.45 (br s, 1 H), 7.40 (dd, *J* = 8.1, 1.9 Hz, 1 H), 7.30 (d, *J* = 3.7 Hz, 1 H), 7.09 (d, *J* = 3.7 Hz, 1 H), 6.83 (d, *J* = 8.0 Hz, 1 H), 3.52 (s, 2 H). LRMS (APCI^+^) calcd for C_12_H_9_INOS 342 (MH^+^), found 342.

***tert-*Butyl (5-(5-(2-oxoindolin-5-yl)thiophen-2-yl)pyridin-3-yl)carbamate (61)**. *tert*-Butyl (5-(4,4,5,5-tetramethyl-1,3,2-dioxaborolan-2-yl)pyridin-3-yl)carbamate and 5-(5-iodothiophen-2-yl)indolin-2-one (**44**) were reacted according to general procedure A. The title compound was isolated as a pale yellow solid (56%). ^1^H NMR [400 MHz, (CD_3_)_2_SO] δ 10.52 (br s, 1 H), 9.71 (br s, 1 H), 8.55 (d, *J* = 2.0, 1 H), 8.48 (d, *J* = 2.2 Hz, 1 H), 8.19 (br s, 1 H), 7.51-7.60 (m, 3 H), 7.44 (d, *J* = 3.8 Hz, 1 H), 6.86 (d, *J* = 8.1 Hz, 1 H), 3.54 (s, 2 H), 1.51 (s, 9 H). LRMS (APCI^+^) calcd for C_22_H_22_N_3_O_3_S 408 (MH^+^), found 408.

**5-(5-(5-Aminopyridin-3-yl)thiophen-2-yl)indolin-2-one (69)**. Deprotection of *tert-*butyl (5-(5-(2-oxoindolin-5-yl)thiophen-2-yl)pyridin-3-yl)carbamate (**61**) according to general procedure E gave the title compound as a cream solid (100%). ^1^H NMR [400 MHz, (CD_3_)_2_SO] δ 10.51 (br s, 1 H), 8.08 (d, *J* = 1.9 Hz, 1 H), 7.86 (d, *J* = 2.4 Hz, 1 H), 7.55 (br s, 1 H), 7.50 (dd, *J* = 8.1, 1.9 Hz, 1 H), 7.44 (d, *J* = 3.8 Hz, 1 H), 7.39 (d, *J* = 3.8 Hz, 1 H), 7.12 (t, *J* = 2.2 Hz, 1 H), 6.86 (d, *J* = 8.1 Hz, 1 H), 5.46 (br s, 2 H), 3.54 (s, 2 H). LRMS (APCI^+^) calcd for C_17_H_14_N_3_OS 308 (MH^+^), found 308.

**2,4-Difluoro-*N*-(5-(5-(2-oxoindolin-5-yl)thiophen-2-yl)pyridin-3-yl)benzenesulfonamides (14)**. 5-(5-(5-Aminopyridin-3-yl)thiophen-2-yl)indolin-2-one (**69**) was reacted with 2,4-difluorobenzenesulfonyl chloride according to general procedure C, and the resulting crude product purified by flash column chromatography (5% MeOH/CH_2_Cl_2_ as eluant) to give the title compound as a pale yellow solid (52%), mp (MeOH/CH_2_Cl_2_) 301-305^o^C. ^1^H NMR [400 MHz, (CD_3_)_2_SO] δ 11.11 (br s, 1 H), 10.53 (br s, 1 H), 8.64 (d, *J* = 2.0 Hz, 1 H), 8.22 (d, *J* = 2.3 Hz, 1 H), 7.96-8.04 (m, 1 H), 7.68 (t, *J* = 2.2 Hz, 1 H), 7.50-7.61 (m, 4 H), 7.44 (d, *J* = 3.8 Hz, 1 H), 7.30 (td, *J* = 8.3, 2.0 Hz, 1 H), 6.87 (d, *J* = 8.1 Hz, 1 H), 3.55 (s, 2 H). LRMS (APCI^+^) calcd for C_23_H_16_F_2_N_3_O_3_S_2_ 484 (MH^+^), found 484.

**5-(5-Iodothiophen-2-yl)-1-methylindolin-2-one (45).** 1-Methyl-5-(thiophen-2-yl)indolin-2-one (**33**) was prepared according to a literature procedure^4^ then iodinated with *N*-iodosuccinimide according to general procedure B. The title compound was isolated as a pale brown solid (97%). ^1^H NMR [400 MHz, (CD_3_)_2_SO] δ 7.49-7.53 (m, 2 H), 7.32 (d, *J* = 3.8 Hz, 1 H), 7.14 (d, *J* = 3.8 Hz, 1 H), 7.00 (d, *J* = 8.7 Hz, 1 H), 3.59 (s, 2 H), 3.13 (s, 3 H). LRMS (APCI^+^) calcd for C_13_H_11_IOS 356 (MH^+^), found 356.

***tert*-Butyl (5-(5-(1-methyl-2-oxoindolin-5-yl)thiophen-2-yl)pyridin-3-yl)carbamate (62)**. *tert*-Butyl (5-(4,4,5,5-tetramethyl-1,3,2-dioxaborolan-2-yl)pyridin-3-yl)carbamate and 5-(5-iodothiophen-2-yl)-1-methylindolin-2-one (**45**) were reacted according to general procedure A. The title compound was isolated as a pale brown solid (91%). ^1^H NMR [400 MHz, (CD_3_)_2_SO] δ 9.71 (br s, 1 H), 8.56 (d, *J* = 2.1 Hz, 1 H), 8.48 (d, *J* = 2.3 Hz, 1 H), 8.21 (br s, 1 H), 7.62-7.66 (m, 2 H), 7.56 (d, *J* = 3.8 Hz, 1 H), 7.48 (d, *J* = 3.8 Hz, 1 H), 7.04 (d, *J* = 8.7 Hz, 1 H), 3.62 (s, 2 H), 3.15 (s, 3 H), 1.51 (s, 9 H). LRMS (APCI^+^) calcd for C_23_H_24_N_3_O_3_S 422 (MH^+^), found 422.

**5-(5-(5-Aminopyridin-3-yl)thiophen-2-yl)-1-methylindolin-2-one (70)**. Deprotection of *tert*-butyl (5-(5-(1-methyl-2-oxoindolin-5-yl)thiophen-2-yl)pyridin-3-yl)carbamate (**62**) according to general procedure E gave the title compound as a pale yellow solid (78%). ^1^H NMR [400 MHz, (CD_3_)_2_SO] δ 8.09 (d, *J* = 2.0 Hz, 1 H), 7.86 (d, *J* = 2.1 Hz, 1 H), 7.58-7.62 (m, 2 H), 7.44 (d, *J* = 3.8 Hz, 1 H), 7.45 (d, *J* = 3.8 Hz, 1 H), 7.13 (t, *J* = 2.3 Hz, 1 H), 7.03 (d, *J* = 8.7 Hz, 1 H), 5.47 (s, 2 H), 3.62 (s, 2 H), 3.15 (s, 3 H). LRMS (APCI^+^) calcd for C_18_H_16_N_3_OS 322 (MH^+^), found 322.

**2,4-Difluoro-*N*-(5-(5-(1-methyl-2-oxoindolin-5-yl)thiophen-2-yl)pyridin-3-yl)benzenesulfonamides (15)**. 5-(5-(5-Aminopyridin-3-yl)thiophen-2-yl)-1-methylindolin-2-one (**70**) was reacted with 2,4-difluorobenzenesulfonyl chloride according to general procedure C, and the resulting crude product purified by flash column chromatography (2% MeOH/CH_2_Cl_2_ as eluant) to give the title compound as a dark yellow solid (93%), mp (MeOH/CH_2_Cl_2_) 268-271^o^C. ^1^H NMR [400 MHz, (CD_3_)_2_SO] δ 11.11 (br s, 1 H), 8.63 (d, *J* = 1.7 Hz, 1 H), 8.21 (d, *J* = 2.4 Hz, 1 H), 4.96-8.03 (m, 1 H), 7.67 (t, *J* = 2.2 Hz, 1 H), 7.61-7.66 (m, 2 H), 7.52-7.59 (m, 2 H), 7.48 (d, *J* = 3.8 Hz, 1 H), 7.30 (td, *J* = 8.3, 2.0 Hz, 1 H), 7.05 (d, *J* = 8.0 Hz, 1 H), 3.62 (s, 2 H), 3.15 (s, 3 H). LRMS (APCI^-^) calcd for C_24_H_16_F_2_N_3_O_3_S_2_ 496 (M-H), found 496.

**5-(5-Bromothiophen-2-yl)-1*H*-indole (46)**. Reaction of 2,5-dibromothiophene and indole-5-boronic acid according to general procedure A gave the title compound as a crystalline yellow solid (51%). ^1^H NMR [400 MHz, (CD_3_)_2_SO] δ 11.22 (br s, 1 H), 7.77 (d, *J* = 1.7 Hz, 1 H), 7.42 (dd, *J* = 8.5, 0.7 Hz, 1 H), 7.39 (t, *J* = 2.7 Hz, 1 H), 7.33 (dd, *J* = 8.5, 1.8 Hz, 1 H), 7.22 (d, *J* = 3.8, 1 H), 7.20 (d, *J* = 3.8 Hz, 1 H), 6.45-6.48 (m, 1 H). LRMS (APCI^+^) calcd for C_12_H_9_BrNS 278, 280 (MH^+^), found 278, 280.

**5-(5-(1*H*-Indol-5-yl)thiophen-2-yl)pyridin-3-amine (52)**. Reaction of 5-(5-bromothiophen-2-yl)-1*H*-indole (**46**) and 3-aminopyridine-5-boronic acid according to general procedure A gave the title compound as an olive-green powder (63%). ^1^H NMR [400 MHz, (CD_3_)_2_SO] δ 11.22 (br s, 1 H), 8.10 (d, *J* = 2.0 Hz, 1 H), 7.85 (d, *J* = 2.3 Hz, 2 H), 7.42-7.47 (m, 3 H), 7.41 (d, *J* = 3.8 Hz, 1 H), 7.39 (t, *J* = 2.7 Hz, 1 H), 7.14 (t, *J* = 2.2 Hz, 1 H), 6.48 (d, *J* = 2.4 Hz, 1 H), 5.46 (br s, 2 H). LRMS (APCI^+^) calcd for C_17_H_14_N_3_S 292 (MH^+^), found 292.

***N*-(5-(5-(1*H*-indol-5-yl)thiophen-2-yl)pyridin-3-yl)-2,4-difluorobenzenesulfonamide (16)**. 5-(5-(1*H*-Indol-5-yl)thiophen-2-yl)pyridin-3-amine (**52**) was reacted with 2,4-difluorobenzenesulfonyl chloride according to general procedure C, and the resulting crude product purified by flash column chromatography (2% MeOH/CH_2_Cl_2_ as eluant) to give the title compound as a dark yellow solid (80%), mp (CH_2_Cl_2_/Et_2_O) 228-231^o^C. ^1^H NMR [400 MHz, (CD_3_)_2_SO] δ 11.23 (br s, 1 H), 11.10 (br s, 1 H), 8.64 (br s, 1 H), 8.21 (br s, 1 H), 7.97-8.04 (m, 1 H), 7.89 (s, 1 H), 7.68 (br s, 1 H), 7.53-7.60 (m, 2 H), 7.43-7.47 (m, 3 H), 7.40 (t, *J* = 2.7 Hz, 1 H), 7.28-7.34 (m, 1 H), 6.48-6.51 (m, 1 H). LRMS (APCI^+^) calcd for C_23_H_16_F_2_N_3_O_2_S_2_ 468 (MH^+^), found 468.

**5-(5-Bromothiophen-2-yl)-1-methyl-1*H*-indole (47)**. Reaction of 2,5-dibromothiophene and 1-methylindole-5-boronic acid according to general procedure A gave the title compound as a pale yellow solid (57%). ^1^H NMR [400 MHz, (CD_3_)_2_SO] δ 7.77 (d, *J* = 1.2 Hz, 1 H), 7.48 (d, *J* = 8.6 Hz, 1 H), 7.40 (dd, *J* = 8.6, 1.8 Hz, 1 H), 7.37 (d, *J* = 3.0 Hz, 1 H), 7.25 (d, *J* = 3.9, 1 H), 7.20 (d, *J* = 3.9 Hz, 1 H), 6.46 (dd, *J* = 3.1, 0.7 Hz, 1 H), 3.80 (s, 3 H). LRMS (APCI^+^) calcd for C_13_H_11_BrNS 292, 294 (MH^+^), found 292, 294.

**5-(5-(1-Methyl-1*H*-indol-5-yl)thiophen-2-yl)pyridin-3-amine (53)**. Reaction of 5-(5-bromothiophen-2-yl)-1-methyl-1*H*-indole (**47**) and 3-aminopyridine-5-boronic acid according to general procedure A gave the title compound as a dark yellow solid (65%). ^1^H NMR [400 MHz, (CD_3_)_2_SO] δ 8.10 (d, *J* = 2.0 Hz, 1 H), 7.84-7.87 (m, 2 H), 7.48-7.51 (m, 2 H), 7.42-7.47 (m, 2 H), 7.37 (d, *J* = 3.0 Hz, 1 H), 7.14 (t, *J* = 2.2 Hz, 1 H), 6.48 (d, *J* = 3.0 Hz, 1 H), 5.49 (br s, 2 H), 3.81 (s, 3 H). LRMS (APCI^+^) calcd for C_18_H_16_N_3_S 306 (MH^+^), found 306.

**2,4-Difluoro-*N*-(5-(5-(1-methyl-1*H*-indol-5-yl)thiophen-2-yl)pyridin-3-yl)benzenesulfonamide (17)**. 5-(5-(1-Methyl-1*H*-indol-5-yl)thiophen-2-yl)pyridin-3-amine (**53**) was reacted with 2,4-difluorobenzenesulfonyl chloride according to general procedure C, and the resulting crude product purified by flash column chromatography (2% MeOH/CH_2_Cl_2_ as eluant) to give the title compound as a pale yellow solid (83%), mp (MeOH/CH_2_Cl_2_) 235-238^o^C. ^1^H NMR [400 MHz, (CD_3_)_2_SO] δ 11.10 (br s, 1 H), 8.66 (d, *J* = 1.8 Hz, 1 H), 8.22 (d, *J* = 2.3 Hz, 1 H), 7.97-8.05 (m, 1 H), 7.89 (t, *J* = 1.1 Hz, 1 H), 7.69 (t, *J* = 2.2 Hz, 1 H), 7.53-7.61 (m, 2 H), 7.50-7.52 (m, 2 H), 7.48 (d, *J* = 3.8 Hz, 1 H), 7.38 (d, *J* = 3.0 Hz, 1 H), 7.28-7.34 (m, 1 H), 6.49 (d, *J* = 3.0 Hz, 1 H), 3.82 (s, 3 H). LRMS (APCI^+^) calcd for C_24_H_18_F_2_N_3_O_2_S_2_ 482 (MH^+^), found 482.

**1-(1-Methyl-5-(thiophen-2-yl)-1*H*-indol-3-yl)ethan-1-one (34)**

A 25 mL round bottom flask was charged with thiophen-2-ylboronic acid (321 mg, 2.51 mmol), 1-(5-bromo-1-methyl-1*H*-indol-3-yl)ethan-1-one (352 mg, 1.40 mmol) and PdCl_2_(dppf) (40.0 mg, 54 μmol. The mixture was suspended in DMF (9 mL) and 2M KHCO_3_ (3 mL), degassed and then heated to 70 °C for 3 h. The reaction was diluted with saturated NH_4_Cl, extracted with CH_2_Cl_2_, dried over MgSO_4_ and concentrated to give a black solid. Chromatography with silica gel eluting with CH_2_Cl_2_/MeOH 98:2 gave the title compound as a cream coloured solid (293 mg, 82%); 1H NMR [400 MHz, CDCl_3_] δ 8.64 (dd, *J =* 1.8, 0.7 Hz, 1 H), 7.69 (s, 1 H), 7.59 (dd, *J =* 8.5, 1.8 Hz, 1 H), 7.37 (dd, *J =* 3.6, 1.2 Hz, 1 H), 7.32 (dd, *J =* 8.5, 0.6 Hz, 1 H), 7.26 (dd, *J =* 5.1, 1.2 Hz, 1 H), 7.09 (dd, *J =* 5.1, 1.2 Hz, 1 H), 3.85 (s, 3 H), 2.53 (s, 3 H). LRMS (APCI^+^) calcd for C_15_H_14_NOS 256 (MH^+^), found 256.

**1-(5-(5-(5-Aminopyridin-3-yl)thiophen-2-yl)-1-methyl-1H-indol-3-yl)ethan-1-one (54).**

1-(1-Methyl-5-(thiophen-2-yl)-1*H*-indol-3-yl)ethan-1-one (**34**) was iodinated with *N*-iodosuccinimide according to general procedure B. The resulting crude iodide, 1-(5-(5-iodothiophen-2-yl)-1-methyl-1H-indol-3-yl)ethan-1-one (**48**) was then used directly in the following Suzuki step where it was reacted with 3-aminopyridine-5-boronic acid according to general procedure A, giving the title compound was as a cream solid (26%). ^1^H NMR [400 MHz, CDCl_3_] δ 8.64 (d, *J =* 1.4 Hz, 1 H), 8.30 (dd, *J =* 4.0, 1.0 Hz, 1 H), 7.98 (dd, *J =* 5.4, 1.8 Hz, 1 H), 7.69 (s, 1 H), 7.56 (dd, *J =* 8.5, 1.9 Hz, 1 H), 7.45 (dd, *J =* 8.1, 1.6 Hz, 1 H), 7.32 (d, *J =* 2.3 Hz, 1 H), 7.21 (d, *J =* 3.6 Hz, 1 H), 6.93 (d, *J =* 8.1 Hz, 1 H), 4.02-3.73 (s-br, 2 H), 3.83 (s, 3 H), 3.33 (s, 3 H); LRMS (APCI^+^) calcd for C_20_H_18_N_3_OS 348 (MH^+^), found: 348.

***N*-(5-(5-(3-Acetyl-1-methyl-1*H*-indol-5-yl)thiophen-2-yl)-pyridin-3-yl)-2,4-difluorobenzenesulfonamide (18).** 1-(5-(5-(5-Aminopyridin-3-yl)thiophen-2-yl)-1-methyl-1H-indol-3-yl)ethan-1-one (**54**) was reacted with 2,4-difluorobenzenesulfonyl chloride according to general procedure C, affording the title compound as an off-white solid (7.5%), mp (MeOH/CH_2_Cl_2_) 241-244°C. ^1^H NMR [400 MHz, (CD_3_)_2_SO] δ 11.1 (br s, 1 H), 8.69 (d, *J* = 2.0 Hz, 1 H), 8.48 (d, *J* = 1.4 Hz, 1 H), 8.39 Hz (s, 1 H), 8.23 (d, *J* = 2.4 Hz, 1 H), 7.98-8.05 (m, 1 H), 7.72 (t, *J* = 2.2, 2.2 Hz, 1 H), 7.52-7.70 (m, 5 H), 7.28-7.34 (m, 1 H), 3.89 (s, 3 H), 2.46 (s, 3 H).

**5-(Furan-2-yl)-2-methylisoindolin-1-one (71).** Reaction of 5-bromoisoindolin-1-one and 2-furanboronic acid according to general procedure A gave the title compound as an off-white solid (66%). ^1^H NMR [400 MHz, (CD_3_)_2_SO] δ 7.90 (s, 1 H), 7.81 (dd, *J* = 9.8, 1.8 Hz, 2 H), 7.68 (d, *J* = 8.0, 1 H), 7.11 (dd, *J* = 3.4, 0.5 Hz, 1 H), 6.65 (dd, *J* = 3.4, 1.8 Hz, 1 H), 4.49 (s, 2 H), 3.08 (s, 3 H). LRMS (APCI^+^) calcd for C_13_H_11_NO_2_ 214 (MH^+^), found 214.

**5-(5-Iodofuran-2-yl)-2-methylisoindolin-1-one (73).** Iodination of 5-(furan-2-yl)-2-methylisoindolin-1-one (**71**) with *N*-iodosuccinimide according to general procedure B gave the title compound as a light-brown solid (82%, crude). This compound was used directly in the next step without further purification. LRMS (APCI^+^) calcd for C_13_H_10_INO_2_ 340 (MH^+^), found 340.

**5-(5-(5-Aminopyridin-3-yl)furan-2-yl)-2-methylisoindolin-1-one (75).** 5-(5-Iodofuran-2-yl)-2-methylisoindolin-1-one (**73**) was reacted with 5-(4,4,5,5-tetramethyl-1,3,2-dioxaborolan-2-yl)pyridin-3-amine (1.2 equivalents) in a sealed tube according to general procedure A. Purification by column chromatography on silica gel (1-3% MeOH/CH_2_Cl_2_) gave the title compound as a brown solid (62%). ^1^H NMR [400 MHz, (CD_3_)_2_SO] δ 8.26 (d, *J* = 1.7 Hz, 1 H), 7.99 (s, 1 H), 7.91 (d, *J* = 1.3 Hz, 1 H), 7.89 (d, *J* = 1.8 Hz, 1 H), 7.72 (d, *J* = 7.9 Hz, 1 H), 7.33 (t, *J* = 2.2 Hz, 1 H), 7.26 (d, *J* = 3.5 Hz, 1 H), 7.10 (d, *J* = 3.6 Hz, 1 H), 5.48 (br s, 2 H), 4.52 (s, 2 H), 3.09 (s, 3 H). LRMS (APCI^+^) calcd for C_18_H_15_N_3_O_2_ 306 (MH^+^), found 306.

**2,4-Difluoro-*N*-(5-(5-(2-methyl-1-oxoisoindolin-5-yl)furan-2-yl)pyridin-3-yl)benzenesulphonamide (19).** 5-(5-(5-Aminopyridin-3-yl)furan-2-yl)-2-methylisoindolin-1-one (**75**) was reacted with benzenesulphonyl chloride according to general procedure C and the desired title compound was given as a beige solid (17%); mp 232-235^o^C. ^1^H NMR [400 MHz, (CD_3_)_2_SO] δ 11.11 (br s, 1 H), 8.65 (s, 1 H), 8.16 (d, *J* = 1.8 Hz, 1 H), 7.96-8.05 (m, 2 H), 7.88 (dd, *J* = 8.0, 1.3 Hz, 1 H), 7.74 (d, *J* = 7.7 Hz, 2 H), 7.49 (t, *J* = 9.5 Hz, 1 H), 7.28 (d, *J* = 3.6 Hz, 1 H), 7.25 (dd, *J* = 8.8, 1.8 Hz, 1 H), 7.20 (d, *J* = 3.5 Hz, 1 H), 4.55 (s, 2 H), 3.10 (s, 3 H). LRMS (APCI^+^) calcd for C_24_H_17_F_2_N_3_O_4_S 483 (MH^+^), found 483. HPLC purity: 98.5%.

**2-Methyl-5-(thiazol-2-yl)isoindolin-1-one (72).** 2-Bromothiazole was reacted with 2-methyl-5-(4,4,5,5-tetramethyl-1,3,2-dioxaborolan-2-yl)isoindolin-1-one according to general procedure A, to give the title compound as a light-brown solid (64%). ^1^H NMR [400 MHz, (CD_3_)_2_SO] δ 8.18 (d, *J* = 0.7 Hz, 1 H), 8.07 (dd, *J* = 7.9, 1.5 Hz, 1 H), 8.00 (d, *J* = 3.2 Hz, 1 H), 7.88 (d, *J* = 3.2 Hz, 1 H), 7.77 (d, *J* = 7.9 Hz, 1 H), 4.54 (s, 2 H), 3.09 (s, 3 H). LRMS (APCI^+^) calcd for C_12_H_10_N_2_OS 231 (MH^+^), found 231.

**5-(5-Iodothiazol-2-yl)-2-methylisoindolin-1-one (74).** 2-Methyl-5-(thiazol-2-yl)isoindolin-1-one (**72**) was iodinated with *N*-iodosuccinimide according to general procedure B. After workup, the crude solid was filtered through a plug of silica gel (1% MeOH/CH_2_Cl_2_), concentrated in vacuo, and recrystallised from 5% MeOH/CH_2_Cl_2_ and hexanes to give the title compound as a light-brown solid (12%). This was used directly in the next step without further purification. ^1^H NMR [400 MHz, (CD_3_)_2_SO] δ 8.13 (s, 1 H), 8.10 (s, 1 H), 8.00 (dd, *J* = 8.0, 1.5 Hz, 1 H), 7.76 (d, *J* = 7.9, 1 H), 4.54 (s, 2 H), 3.09 (s, 3 H). LRMS (APCI^+^) calcd for C_12_H_9_IN_2_OS 357 (MH^+^), found 357.

**5-(5-(5-Aminopyridin-3-yl)thiazol-2-yl)-2-methylisoindolin-1-one (76).** 5-(5-Iodothiazol-2-yl)-2-methylisoindolin-1-one (**74**) was reacted with 5-(4,4,5,5-tetramethyl-1,3,2-dioxaborolan-2-yl)pyridin-3-amine according to general procedure A. Purification by column chromatography on silica gel (1-5% MeOH/CH_2_Cl_2_) gave the title compound as a brown solid (41%). ^1^H NMR [400 MHz, (CD_3_)_2_SO] δ 8.34 (s, 1 H), 8.20 (s, 1 H), 8.14 (s, 1 H), 8.09 (d, *J* = 7.4 Hz, 1 H), 7.95 (s, 1 H), 7.80 (d, *J* = 8.04 Hz, 1 H), 7.18 (s, 1 H), 4.56 (s, 2 H), 3.10 (s, 3 H). LRMS (APCI^+^) calcd for C_17_H_14_N_4_OS 323 (MH^+^), found 323.

**2,4-Difluoro-*N*-(5-(2-(2-methyl-1-oxoisoindolin-5-yl)thiazol-5-yl)pyridin-3-yl) benzenesulphonamide (20).** 5-(5-(5-Aminopyridin-3-yl)thiazol-2-yl)-2-methylisoindolin-1-one (**76**) was reacted with 2,4-difluorobenzenesulphonyl chloride according to general procedure C, and the desired title compound was given as a beige solid (16%), mp >300^o^C. ^1^H NMR [400 MHz, (CD_3_)_2_SO] δ 11.21 (br s, 1 H), 8.74 (d, *J* = 2.0 Hz, 1 H), 8.46 (s, 1 H), 8.31 (d, *J* = 2.4 Hz, 1 H), 8.22 (d, *J* = 0.7 Hz, 1 H), 8.11 (dd, *J* = 7.9, 1.4Hz, 1 H), 7.98-8.07 (m, 1 H), 7.80 (d, *J* = 7.9 Hz, 1 H), 7.76 (t, *J* = 2.2 Hz, 1 H), 7.53-7.62 (m, 1 H), 7.31 (dt, *J* = 8.2, 2.2 Hz, 1 H), 4.56 (s, 2 H), 3.11 (s, 3 H).

**5-(4-Bromothiazol-2-yl)-2-methylisoindolin-1-one (77).** 2,4-Dibromothiazole was reacted with 2-methyl-5-(4,4,5,5-tetramethyl-1,3,2-dioxaborolan-2-yl)isoindolin-1-one according to general procedure A. Purification by column chromatography on silica gel (2% MeOH/CH_2_Cl_2_) gave the title product as a beige solid (53%) ^1^H NMR [400 MHz, (CD_3_)_2_SO] δ 8.18 (dd, *J =* 1.5, 0.7 Hz, 1 H), 8.03 (dd, *J =* 7.9 Hz, 1.6 Hz, 1 H), 7.99 (s, 1 H), 7.77 (d, *J =* 7.9 Hz, 1 H), 4.54 (s, 2 H), 3.09 (s, 3 H). LRMS (APCI^+^) calcd for C_13_H_9_BrN_2_OS 311 (MH^+^), found 311.

**3-Bromo-*N*-(ethoxymethyl)-4-methyl-*N*-(pyridin-2-yl)benzenesulfonamide (78).** 2,4-Difluorobenzenesulfonyl chloride and 5-bromopyridin-3-amine were reacted according to general procedure C. Without any further purification, to the resulting crude solid and (chloromethoxy)ethane (319 mg, 3.36 mmol) in DMF at room temperature was added a 60% w/w dispersion of NaH in mineral oil (130 mg, 3.36 mmol) and the mixture was stirred for 1 h. After quenching with water the mixture was extracted with CH_2_Cl_2_, dried with MgSO_4_ and evaporated to give a solid which was purified by column chromatography on silica gel (3:1 hexanes/EtOAc) gave gave the product as colourless oil which solidified to a crystalline white solid upon standing (558 mg, 61%). ^1^H NMR [400 MHz, CDCl_3_] δ 8.61 (d, *J =* 8.6 Hz, 1 H), 8.33 (d, *J =* 2.2 Hz, 1 H), 7.80 (dd, *J =* 2.1, 2.1 Hz, 1 H), 7.75-7.69 (m, 1 H), 7.00-6.92 (m, 1 H), 5.14 (s, 2 H), 3.76 (q, *J =* 7.0 Hz, 2 H), 1.20 (t, *J =* 7.0 Hz, 3 H). LRMS (APCI^+^) calcd for C_14_H_14_BrF_2_N_2_O_3_S 407 (MH^+^) , found: 407.

**2,4-difluoro-*N*-(5-(2-(2-methyl-1-oxoisoindolin-5-yl)thiazol-4-yl)pyridin-3-yl)benzenesulfonamides (21)**. *N*-(5-bromopyridin-3-yl)-*N*-(ethoxymethyl)-2,4-difluorobenzenesulfonamide (**78**) was converted to the corresponding pinacol ester according to general procedure G. The crude boronate was then reacted with 5-(4-bromothiazol-2-yl)-2-methylisoindolin-1-one (**77**) using a Suzuki reaction as described in general procedure A. After extraction of the reaction mixture with EtOAc and evaporation, the crude intermediate was subjected to a one-pot deprotection and conversion to the sodium salt as follows; the solid was taken up in a 1:1 solution of 3 M HCl and dioxane and then heated to reflux for 1 h. Upon cooling the white precipitate, consisting of essentially pure sulfonamide, was filtered and taken up in EtOH. Precipitation of the sodium salt was accomplished by slow addition 2 M NaOH to give the title product as a yellow precipitate (33.6 mg, 9%, 4 steps); mp 232-236^o^C. ^1^H NMR [400 MHz, (CD_3_)_2_SO] δ 8.37 (d, *J =* 2.0 Hz, 1 H), 8.23 (br s, 1 H), 8.11-8.08 (m, 2 H), 7.98 (d, *J =* 2.0 Hz, 1 H), 7.88 (ddd, *J =* 8.5, 8.5, 6.9 Hz, 1 H), 7.80 (d, *J =* 7.9 Hz, 1 H), 7.73-7.72 (m, 1 H), 7.18 (ddd, *J =* 9.7, 9.7, 2.5 Hz, 1 H), 7.07 (ddd, *J =* 8.4, 8.4, 2.1 Hz, 1 H), 4.59 (s, 2 H), 3.11 (s, 3 H). HRMS (ESI+) calcd for C_23_H_16_F_2_N_4_NaO_3_S_2_ 521.0530 (MH^+^), found 521.0508.

**5-(6-Aminopyridin-3-yl)-2-methylisoindolin-1-one (79).** 5-Bromo-2-methylisoindolin-1-one was reacted with 5-(4,4,5,5-tetramethyl-1,3,2-dioxaborolan-2-yl)pyridin-2-amine according to general procedure A. Purification by column chromatography on silica gel (1-3% MeOH/CH_2_Cl_2_) gave the title compound as a brown solid (68%). ^1^HNMR [400 MHz, (CD_3_)_2_SO] δ 8.30 (s, 1 H), 7.80 (s, 2 H), 7.60 (s, 2 H), 6.54 (d, *J* = 8.4 Hz, 1 H), 6.17 (s, 2 H), 4.47 (s, 2 H), 3.08 (s, 3 H). LRMS (APCI^+^) calcd for C_14_H_13_N_3_O 240 (MH^+^), found 240.

**5-(6-Bromopyridin-3-yl)-2-methylisoindolin-1-one (80).** 5-(6-Aminopyridin-3-yl)-2-methylisoindolin-1-one (**79**) (300 mg, 1.26 mmol) was dissolved slowly with constant stirring in 47% HBr solution (5 mL) and cooled to -10^o^C. A solution of NaNO_2_ (277 mg, 3.26 mmol) in H_2_O (3 mL) was added slowly to the reaction mixture followed by drop-wise addition of bromine (0.2 mL, 3.77 mmol). The reaction mixture was allowed to warm to room temperature and left to stir until the starting material had been consumed (TLC). The reaction mixture was neutralised with cold aqueous Na_2_CO_3_ and extracted in CH_2_Cl_2_ (3x50 mL). The combined organic extracts were washed with brine (1x50 mL), dried (Na_2_SO_4_), filtered through a plug of silica gel, and concentrated *in vacuo* to give the title compound as a dark-brown solid (133 mg, 35%). This was used directly in the next step without further purification. LRMS (APCI^+^) calcd for C_14_H_11_BrN_2_O 304 (MH^+^), found 304.

***tert*-Butyl (5-(2-methyl-1-oxoisoindolin-5-yl)-[2,3’-bipyridin]-5’-yl)carbamate (81).** 5-(6-Bromopyridin-3-yl)-2-methylisoindolin-1-one (**80**) was reacted with *tert*-butyl (5-(4,4,5,5-tetramethyl-1,3,2-dioxaborolan-2-yl)pyridin-3-yl)carbamate according to general procedure A. Purification by column chromatography on silica gel (1-5% MeOH/CH_2_Cl_2_) gave the title compound as an off-white solid (55%). ^1^H NMR [400 MHz, (CD_3_)_2_SO] δ 9.75 (br s, 1 H), 9.11 (d, *J* = 1.9 Hz, 1 H), 8.93 (d, *J* = 1.9 Hz, 1 H), 8.72 (s, 1 H), 8.65 (d, *J* = 2.4 Hz, 1 H), 8.30 (dd, *J* = 8.4, 2.4 Hz, 1 H), 8.13 (d, *J* = 8.1 Hz, 1 H),8.03 (s, 1 H), 7.91 (dd, *J* = 8.0, 1.2Hz, 1 H), 7.80 (d, *J* = 7.9 Hz, 1 H), 4.56 (s, 2 H), 3.11 (s, 3 H), 1.52 (s, 9 H). LRMS (APCI^+^) calcd for C_24_H_24_N_4_O_3_ 418 (MH^+^), found 418.

**5-(5’-Amino-[2,3’-bipyridin]-5-yl)-2-methylisoindolin-1-one (81).** Deprotection of *tert*-butyl (5-(2-methyl-1-oxoisoindolin-5-yl)-[2,3’-bipyridin]-5’-yl)carbamate (**82**) according to general procedure E gave the title compound as an off-white solid (86%). ^1^H NMR [400 MHz, (CD_3_)_2_SO] δ 9.05 (dd, *J* = 2.4, 0.6 Hz, 1 H), 8.48 (d, *J* = 1.9 Hz, 1 H), 8.25 (dd, *J* = 8.4, 2.5 Hz, 1 H), 8.01-8.06 (m, 2 H), 8.00 (d, *J* = 2.6 Hz, 1 H), 7.90 (dd, *J* = 7.9, 1.5 Hz, 1 H), 7.79 (d, *J* = 7.9 Hz, 1 H), 7.69 (t, *J* = 2.3 Hz, 1 H), 5.48 (br s, 2 H), 4.55 (s, 2 H), 3.11 (s, 3 H). LRMS (APCI^+^) calcd for C_19_H_16_N_4_O 317 (MH^+^), found 317.

**2,4-Difluoro-*N*-(5-(2-methyl-1-oxoisoindolin-5-yl)-[2,3’-bipyridin]-5’-yl)benzenesulphonamide (22).** 5-(5’-Amino-[2,3’-bipyridin]-5-yl)-2-methylisoindolin-1-one (**82**) was reacted with 2,4-difluorobenzenesulphonyl chloride according to general procedure C, and the desired title compound was given as a pink solid (62%), mp 278-282^o^C. ^1^H NMR [400 MHz, (CD_3_)_2_SO] δ 11.12 (br s, 1 H), 9.10 (d, *J* = 2.1 Hz, 1 H), 9.05 (d, *J* = 1.7 Hz, 1 H), 8.41 (d, *J* = 2.4 Hz, 1 H), 8.25-8.34 (m, 2 H), 8.13 (d, *J* = 8.3 Hz, 1 H), 8.04 (s, 1 H), 7.93-8.01 (m, 1 H), 7.90 (d, *J* = 8.6 Hz, 1 H), 7.79 (d, *J* = 7.9 Hz, 1 H), 7.56 (dt, *J* = 8.4, 2.4 Hz, 1 H), 7.29 (dt, *J* = 8.4, 2.1 Hz, 1 H), 4.55 (s, 2 H), 3.11 (s, 3 H).

**1.1.1. Table 1: Elemental analysis results for target compounds**

| **Compound** |  | **Calcd** | | | **Found** | | |
| --- | --- | --- | --- | --- | --- | --- | --- |
|  |  | **C** | **H** | **N** | **C** | **H** | **N** |
| **5** | C_24_H_17_F_2_N_3_O_3_S | 57.9 | 3.4 | 8.5 | 58.1 | 3.4 | 8.3 |
| **5.Na** | C_24_H_16_F_2_N_3_NaO_3_S_2_.H_2_O | 53.6 | 3.4 | 7.8 | 53.7 | 3.4 | 7.7 |
| **6** | C_23_H_15_F_2_N_3_O_3_S_2_.0.25H_2_O | 56.6 | 3.2 | 8.6 | 56.8 | 3.1 | 8.5 |
| **7** | C_23_H_15_F_2_N_3_O_3_S_2_ | 57.1 | 3.1 | 8.7 | 56.9 | 3.3 | 8.5 |
| **8** | C_24_H_17_F_2_N_3_O_3_S_2_ | 57.9 | 3.4 | 8.5 | 58.1 | 3.5 | 8.5 |
| **10** | C_25_H_19_F_2_N_3_O_3_S_2_ | 58.7 | 3.7 | 8.2 | 58.9 | 3.7 | 8.2 |
| **11** | C_24_H_17_F_2_N_3_O_3_S_2_.H_2_O | 55.9 | 3.7 | 8.2 | 55.9 | 3.4 | 8.0 |
| **12** | C_25_H_19_F_2_N_3_O_3_S_2_ | 58.7 | 3.7 | 8.2 | 59.0 | 3.7 | 8.1 |
| **13** | C_25_H_19_F_2_N_3_O_3_S_2_ | 58.7 | 3.7 | 8.2 | 59.0 | 3.7 | 8.2 |
| **14** | C_23_H_15_F_2_N_3_O_3_S_2._0.25H_2_O | 56.6 | 3.2 | 8.6 | 56.5 | 3.0 | 8.5 |
| **15** | C_24_H_17_F_2_N_3_O_3_S_2_ | 57.9 | 3.4 | 8.5 | 57.9 | 3.3 | 8.4 |
| **16** | C_23_H_15_F_2_N_3_O_2_S_2_ | 59.1 | 3.2 | 9.0 | 59.2 | 3.2 | 8.8 |
| **17** | C_24_H_17_F_2_N_3_O_2_S_2_ | 59.9 | 3.6 | 8.7 | 60.0 | 3.5 | 8.6 |
| **18** | C_26_H_19_F_2_N_3_O_3_S_2_ | 59.6 | 3.7 | 8.0 | 59.5 | 3.7 | 7.9 |
| **19** | C_24_H_17_F_2_N_3_O_4_S.0.5H_2_O | 58.8 | 3.7 | 8.6 | 58.9 | 3.7 | 8.5 |
| **21** | C_23_H_16_F_2_N_4_O_3_S_2_ | 55.4 | 3.2 | 11.2 | 55.3 | 3.2 | 11.1 |
| **22** | C_25_H_18_F_2_N_4_O_3_S.0.75H_2_O | 59.3 | 3.9 | 11.1 | 59.5 | 3.8 | 10.9 |

**1.1.2 Table 3: HRMS and HPLC results for target compounds**

| **Compound** | **HRMS** | | | **HPLC** |
| --- | --- | --- | --- | --- |
|  | **Formula** | **Calcd.** | **Found** |  |
| **9** | C_24_H_17_F_2_N_3_O_3_S_2_ (MH^+^) | 498.0752 | 498.0753 | 99.7% |
| **20** | C_23_H_17_F_2_N_4_O_3_S_2_ (MH^+^) | 499.0705 | 499.0710 | 99.5% |

**1.2. Biology**

**1.2.1. Inhibition of perforin-mediated lysis of Jurkat cells**

The ability of the compounds to inhibit the lysis of nucleated (Jurkat T lymphoma) cells in the presence of 0.1% BSA, as measured by release of ^51^Cr was measured. Jurkat target cells were labelled by incubation in medium with 100 μCi ^51^Cr for one hour. The cells were then washed three times to remove unincorporated isotope and re-suspended at 1 x 10^5^ cells per mL in RPMI buffer supplemented with 0.1% BSA. Each test compound was pre-incubated to concentrations of 20, 10, 5, 2.5 and 1.25 μM with recombinant perforin for 30 min with DMSO as a negative control. ^51^Cr labelled Jurkat cells were then added and cells were incubated at 37 °C for 4 h. The supernatant was collected and assessed for its radioactive content on a gamma counter (Wallac Wizard 1470 automatic gamma counter). Each data point was performed in triplicate and an IC_50_ was calculated from the range of concentrations described to above.

**1.2.2. KHYG-1 cytotoxicity assay**

KHYG-1 cells are washed and re-suspended in RPMI/0.1% BSA at 16 x 10^5^ cells/mL and 100 µL of the cell suspension is dispensed to each well of a 96-well V-bottom plate. Test compounds are then added (50 µL) at a final concentration of 20µM and incubated at RT for 20 min. ^51^Cr-labelled K562 leukemia target cells (50 µL, 2 x 10^5^ cells/mL) are then added to each well and incubated at 37 ºC for 4 hours. ^51^Cr release is assayed using a Skatron Harvesting Press and radioactivity estimated on a Wallac Wizard 1470 Automatic Gamma counter (Turku, Finland). The inhibitory function is then determined by identifying the number of untreated or inhibitor treated effector cells required to kill the same number of targets. The percent inhibition is calculated by the formula:

(*X*)

(16)

*x* 100

100-

Where x is the point the inhibitor intersects with the DMSO on a curve. In the example shown (Fig 1) “*x*” is the x-intercept corresponding to the point on the DMSO control curve that yields the same level of ^51^Cr release as the test compound at an E/T ratio of 16:1.


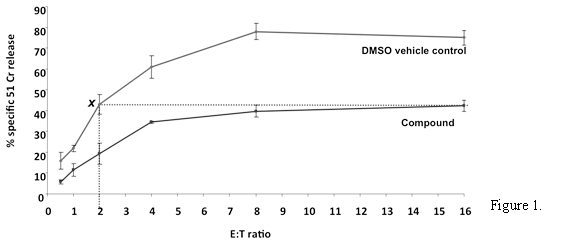


**1.2.3. Toxicity to KHYG-1 NK cells**

The toxicity assay was carried out in exactly the same manner as the killing assay above, but instead of adding the labelled K562 target cells, 100 μL of RPMI 0.1% BSA was added. Cells were incubated for 4 h at 37 °C and then washed ×3 in RPMI + 0.1% BSA. Cells were then re-suspended in 200 μL of complete medium and incubated for 18−24 h at 37 °C. Trypan blue was added to each well. Viable (clear) cells and total (clear + blue) cells were counted, and the percentage of viable cells was calculated compared to DMSO treated cell control (% viability).

**1.2.4. PI3K-α Counterscreening**

Compounds were evaluated for their ability to inhibit the Class I PI3-kinase enzyme p110α/p85. The assays were performed using recombinant PI3-kinase and phosphatidylinositol as a substrate and [^33^P]-ATP tracer with a final ATP concentration of 10 mM, as described previously.^5,6^ IC_50_ data is the average of at least two independent determinations.

**2. References**

1. Ortwine, D. F.; Malone, T. C.; Bigge, C. F.; Drummond, J. T.; Humblet, C.; Johnson, G.; Pinter, G. W. *J. Med. Chem.* **1992**, *35*, 1345-70.

2. Wang, X. –J.; Tan, J.; Grozinger, K. *Tetrahedron Lett.* **1998**, *39*, 6609-6612.

3. Kobayashi, J.; Nakamura, T.; Muranaka, H.; Ishikawa, T.; Tamai, T.; Akahane, S. WO2005040093.

4. Prandi, C.; Occhiato, E. G.; Tabasso, S.; Bonfante, P.; Novero, M.; Scarpi, D.; Bova, M. E.; Miletto, I. *Eur. J. Org. Chem*., **2011**, 3781-3793.

5. Chaussade, C.; Rewcastle, G. W.; Kendall, J. D.; Denny, W. A.; Cho, K. H. Y.; Grønning, L. M.; Chong, M. L.; Anagnostou, S. H.; Jackson, S. P; Daniele, N.; Shepherd, P. R. *Biochem. J*. **2007**, *404*, 449-458.

6. Kendall, J. D.; Rewcastle, G. W.; Frederick, R.; Mawson, C.; Denny, W. A.; Marshall, E. S.; Baguley, B. C.; Chaussade, C. M.; Jackson, S. P.; Shepherd, P. R. *Bioorg. Med. Chem.* **2007**, *15*, 7677-7687.
